# Relative efficacy of different single and combined conservative interventions for adolescent idiopathic scoliosis: a systematic review and network meta-analysis

**DOI:** 10.3389/fpubh.2026.1860459

**Published:** 2026-10-14

**Authors:** Yuanyuan Cao, Meng Tao, Jiachen Shu, Jun Xia, Weibing Wu

**Affiliations:** 1 School of Exercise and Health, Shanghai University of Sport, Shanghai, China; 2 Shanghai Educational Center of Science & Art, Shanghai, China

**Keywords:** adolescent idiopathic scoliosis, combined intervention, conservative intervention, network meta-analysis, Schroth exercise, systematic review

## Abstract

**Objective:**

To systematically evaluate the relative efficacy of different single and combined conservative interventions for adolescent idiopathic scoliosis (AIS), and to provide evidence-based support for individualized conservative treatment strategies in clinical practice.

**Methods:**

Randomized controlled trials on conservative interventions for AIS were retrieved from PubMed, Embase, Web of Science, Scopus, and the Cochrane Library from database inception to December 31, 2025. Two reviewers independently performed study selection, data extraction, and risk-of-bias assessment. A network meta-analysis was conducted using Stata 18.0. The main outcomes included Cobb angle, angle of trunk rotation (ATR), and SRS-22 score. The surface under the cumulative ranking curve (SUCRA) was used to rank different interventions.

**Results:**

A total of 26 randomized controlled trials were included, involving 1,384 patients with AIS and 59 study arms. The interventions included Schroth exercises, orthotic treatment, physiotherapeutic scoliosis-specific exercises (PSSE), Scientific Exercises Approach to Scoliosis (SEAS), core stabilization exercises, Pilates, and their combined interventions. The results showed that, for Cobb angle, Schroth exercises combined with hippotherapy, orthotic treatment combined with SEAS, and orthotic treatment alone ranked relatively high; for ATR, Schroth exercises combined with suspension training, Schroth exercises alone, and routine exercise ranked relatively high; for SRS-22 score, Schroth exercises combined with suspension training, Schroth exercises alone, and routine exercise showed relatively high ranking probabilities. Pairwise comparisons showed that Schroth exercises were superior to proprioceptive neuromuscular facilitation (PNF) for ATR. According to the GRADE assessment, the certainty of evidence was moderate for Cobb angle and SRS-22, and low for ATR.

**Conclusion:**

Both single and combined conservative interventions showed certain therapeutic value for AIS. Some combined interventions demonstrated higher relative efficacy probabilities in improving Cobb angle, ATR, and quality of life. Schroth-based interventions were relatively well-represented in the eligible RCT network and showed favorable ranking probabilities for some outcomes; however, these findings should not be extrapolated to all PSSE approaches.

**Systematic review registration:**

https://www.crd.york.ac.uk/PROSPERO/view/CRD420261369973, identifier: CRD420261369973.

## 1 Introduction

Adolescent idiopathic scoliosis (AIS) is a three-dimensional structural spinal deformity characterized by lateral curvature accompanied by vertebral rotation, primarily occurring during adolescence. A Cobb angle ≥10° is commonly used as the diagnostic criterion (1, 2). With a prevalence of approximately 2%−4%, AIS is one of the most common spinal deformities in adolescents (3, 4). It not only affects appearance and posture but may also lead to long-term adverse effects on cardiopulmonary function, motor performance, and psychological health. Therefore, early identification and intervention are essential for delaying disease progression (5, 6).

According to current guidelines (7), conservative treatment should be prioritized for patients with mild to moderate AIS. Single conservative interventions, such as orthotic treatment (8, 9), physiotherapeutic scoliosis-specific exercises (PSSE) (10), Pilates (11), and core stabilization training (12), can slow curve progression and improve function by enhancing postural control, muscular balance, and spinal stability. In this context, PSSE refers to a broad category of scoliosis-specific exercise approaches, including Schroth, SEAS, BSPTS, DoboMed, FITS, and the Side-Shift Method, rather than to one uniform intervention.

In recent years, conservative treatment has gradually shifted from single interventions to multimodal combined interventions (7, 13). By integrating different therapeutic mechanisms, combined interventions aim to more comprehensively control curve progression and improve functional status (14). Different exercise therapies target distinct aspects, including respiratory regulation, postural reconstruction, and core stability, which may complement each other and produce synergistic effects (15, 16).

However, current studies have not reached consistent conclusions regarding the relative efficacy of single vs. combined interventions. Most randomized controlled trials are limited to pairwise comparisons, and substantial heterogeneity in intervention types and outcome measures makes systematic comparisons difficult (17). Traditional meta-analysis is limited in synthesizing such complex evidence. In contrast, network meta-analysis (NMA) can combine direct and indirect evidence, allowing simultaneous comparisons and ranking of multiple interventions (18, 19). Therefore, a systematic evaluation based on this method is necessary.

Meanwhile, efficacy assessment should include both structural and functional outcomes. The Cobb angle is the radiographic gold standard for evaluating scoliosis severity, while the angle of trunk rotation (ATR) reflects vertebral rotation and external trunk deformity (20). In addition, the Scoliosis Research Society-22 (SRS-22) questionnaire evaluates patient-reported outcomes, including function, pain, mental health, self-image, and treatment satisfaction (21). Accordingly, this study established a comprehensive evaluation system covering Cobb angle, ATR, and quality of life.

Based on this background, the present study systematically reviewed randomized controlled trials and performed a network meta-analysis to compare the relative efficacy of different single and combined conservative interventions for adolescent idiopathic scoliosis. Outcomes included Cobb angle, angle of trunk rotation, and quality of life. By generating probabilistic rankings of intervention strategies, this study aimed to provide evidence-based guidance for the conservative management of AIS.

## 2 Methods

### 2.1 Search strategy

This study was conducted in accordance with a prespecified protocol. Two reviewers independently performed the literature search and recorded the search results. The reviewers worked independently throughout the process, and any disagreements were resolved through discussion; when necessary, a third reviewer was consulted for adjudication. A systematic search was conducted in PubMed, Embase, Web of Science, Scopus, and the Cochrane Library from database inception to December 31, 2025. To maximize the comprehensiveness of the search and minimize the risk of missing relevant studies, the reference lists of included studies and relevant systematic reviews were also manually screened, and clinical trial registries were additionally searched.

The search strategy was developed according to the PICO framework of this study, combining controlled vocabulary terms and free-text terms, and was adapted as appropriate to the indexing systems and search rules of each database. The search terms covered three main domains: (1) population: adolescent idiopathic scoliosis, including terms such as “adolescent idiopathic scoliosis” and “AIS”; (2) interventions: single or combined conservative interventions, including terms such as “exercise,” “physical activity,” “orthotic treatment,” “Schroth,” “SEAS,” “physiotherapeutic scoliosis-specific exercises,” “Pilates,” “core stabilization,” and “non-surgical treatment”; and (3) study design: randomized controlled trials, including terms such as “randomized controlled trial” and “RCT.” Search fields included titles, abstracts, keywords, and corresponding controlled vocabulary fields. The complete database-specific and reproducible search strategies are provided in the Supplementary Appendix, including the exact controlled-vocabulary terms, free-text terms, Boolean operators, truncation or wildcard syntax, search fields, filters, date limits, and RCT search filters used for each database.

Given that this study was designed as a network meta-analysis, prespecified principles were established for the classification of interventions and the definition of treatment nodes before study selection and data extraction. Interventions with similar names but clearly different core therapeutic components, modes of delivery, or clinical meanings were not merged. Combined interventions were defined as separate nodes according to their main components, in order to maximize the clinical interpretability of the network structure and the transitivity of indirect comparisons. To improve reproducibility of treatment-node classification, conservative interventions were operationally defined before study selection. In particular, Schroth exercises were classified only when the intervention explicitly involved three-dimensional spinal correction, rotational angular breathing, and postural awareness training; orthotic treatment was classified only when a rigid orthotic treatment protocol with a prescribed wear time of at least 16 h per day was used; and combined interventions were classified as separate nodes only when two or more eligible conservative treatments were delivered concurrently and each component met its minimum effective dose. No language restrictions were applied. The design, conduct, and reporting of this systematic review and network meta-analysis followed the PRISMA 2020 statement, with consideration of relevant reporting recommendations for network meta-analysis. The study protocol was registered in PROSPERO (registration number: CRD420261369973).

### 2.2 Eligibility criteria

The inclusion and exclusion criteria were defined according to the PICOS framework. Inclusion criteria were as follows: (1) Participants: adolescents with a diagnosis of adolescent idiopathic scoliosis (AIS) confirmed by radiographic examination, with a Cobb angle of ≥10°. Studies were eligible only when participants were described as having idiopathic scoliosis during adolescence; studies involving other clearly defined scoliosis etiologies were not considered eligible. (2) Study design: randomized controlled trials (RCTs). (3) Interventions: single or combined conservative interventions. For the purpose of this review, Schroth exercises were defined as a standardized scoliosis-specific exercise program based on three-dimensional spinal auto-correction, rotational angular breathing, and postural awareness training. Orthotic treatment was defined as treatment with a rigid spinal orthosis (e.g., Boston brace, Charleston brace) worn for at least 16 h per day. Combined interventions were defined as the concurrent use of two or more eligible conservative treatments, with each component reaching its minimum effective dose as prespecified by the study or judged according to commonly accepted clinical practice. Because physiotherapeutic scoliosis-specific exercises (PSSE) represent an umbrella category rather than a single uniform intervention, treatment nodes were defined using prespecified clinical criteria. Named PSSE approaches, such as Schroth and SEAS, were treated as separate nodes only when the original study explicitly identified the approach and provided sufficient information to confirm its defining therapeutic principles. For example, Schroth exercise was classified separately when the intervention included characteristic components such as three-dimensional spinal auto-correction, rotational angular breathing, and postural awareness or stabilization training. Interventions were combined into the same node when they shared the same primary therapeutic objective, core therapeutic components, and clinically comparable mode of delivery, whereas they were separated when clinically meaningful differences existed in the principal corrective strategy, core exercise components, or the addition of a distinct therapeutic component. The generic PSSE node was reserved for interventions described only as PSSE or scoliosis-specific exercise when the available information was insufficient to assign them reliably to a specific named PSSE school; therefore, interventions classified as Schroth or SEAS were not simultaneously included in the generic PSSE node. Minor differences in treatment frequency, session duration, or supervision alone were not considered sufficient to create a separate node. (4) Comparators: other single conservative interventions, combined conservative interventions, usual care, or routine rehabilitation. (5) Outcomes: at least one of the following outcomes was reported: Cobb angle, angle of trunk rotation (ATR), or SRS-22 score. (6) Data availability: sufficient data were available for effect size calculation, including or allowing derivation of sample size, mean values, and standard deviations. For studies with multiple eligible intervention arms, treatment arms were incorporated into the network meta-analysis according to the prespecified principles for treatment node classification.

Exclusion criteria were as follows: (1) non-randomized controlled studies; (2) non-original studies, including reviews, case reports, conference abstracts, study protocols, and dissertations; (3) studies in which participants were not radiographically confirmed AIS patients, or in which the sample included congenital scoliosis, neuromuscular scoliosis, neurofibromatosis-associated scoliosis, syndromic scoliosis, or other clearly defined non-idiopathic scoliosis types; (4) studies in which surgery, postoperative rehabilitation, or other non-conservative treatments were the main intervention; (5) studies that did not report the outcomes of interest, or had incomplete data that precluded extraction or calculation of valid effect sizes; (6) duplicate publications. In cases of duplicate publication, only the report with the most complete information or the most recent data was included.

### 2.3 Data extraction

All records identified through the search were imported into EndNote software for management, and duplicate records were removed. Two reviewers independently screened the studies according to the prespecified inclusion and exclusion criteria. Titles and abstracts were first screened for initial eligibility, followed by full-text assessment of potentially eligible studies. Any disagreements between the two reviewers were resolved through discussion, and when necessary, a third reviewer was consulted for adjudication.

Data were independently extracted by two reviewers using a standardized data extraction form. The extracted information included: (1) general study characteristics: first author, year of publication, and country or region in which the study was conducted; (2) baseline characteristics of participants: sample size, age, sex, body mass index, and other available baseline data; (3) intervention characteristics: type of intervention in the experimental and control groups, intervention duration, frequency, duration of each session, diagnostic criteria used for AIS, baseline Cobb angle, radiographic confirmation, brace type, prescribed daily brace wear time, whether the intervention met the prespecified operational definition of Schroth exercises or orthotic treatment, and whether each component of a combined intervention reached the minimum effective dose; (4) outcome measures: Cobb angle, ATR, and SRS-22 score; and (5) methodological information: key items required for risk-of-bias assessment, such as random sequence generation, allocation concealment, implementation of blinding, completeness of outcome data, and selective reporting.

For multi-arm trials, intervention arms were organized and classified according to the prespecified principles for treatment node definition before being included in the analysis. Any discrepancies arising during data extraction were resolved through discussion between the two reviewers, and if necessary, by consultation with a third reviewer.

### 2.4 Risk of bias assessment and certainty of evidence evaluation

Two reviewers independently assessed the risk of bias of the included studies. Any disagreements were resolved through discussion, and if necessary, a third reviewer was consulted for adjudication. For randomized controlled trials, risk of bias was assessed using the Cochrane risk-of-bias assessment tool (RoB 1) recommended in the Cochrane Handbook, with the assistance of Review Manager 5.4 (The Cochrane Collaboration, Copenhagen, Denmark) software. The assessment domains included random sequence generation, allocation concealment, blinding of participants and personnel, blinding of outcome assessment, completeness of outcome data, selective reporting, and other potential sources of bias. Each domain was judged as having a “low risk,” “high risk,” or “unclear risk” of bias, and an overall judgment of risk of bias was made for each included study on this basis.

In addition, the certainty of evidence for the main outcomes was evaluated using the GRADE approach. The assessment considered study limitations, inconsistency, indirectness, imprecision, and publication bias. The certainty of evidence was categorized into four levels: high, moderate, low, and very low. The evaluation was performed using GRADEpro GDT software.

### 2.5 Statistical analysis

Network meta-analysis was performed using Stata 18.0 (StataCorp LLC, College Station, TX, USA) software. As all outcomes were continuous variables measured on the same scale, weighted mean differences (WMDs) with corresponding 95% confidence intervals (CIs) were used as effect size estimates. The network meta-analysis combined both direct and indirect evidence to compare the relative efficacy of different single and combined conservative interventions for adolescent idiopathic scoliosis.

A network plot was constructed to illustrate the comparative relationships among interventions. In the network graph, each node represents an intervention, the size of the node is proportional to the total sample size for that intervention, and the thickness of the connecting lines reflects the number of studies providing direct comparisons between two interventions. Considering the potential clinical and methodological heterogeneity across studies in terms of intervention delivery, treatment duration, and follow-up time, a random-effects model was used for the analysis.

Local inconsistency was assessed using the node-splitting method to evaluate the agreement between direct and indirect evidence. When no significant inconsistency was detected, the consistency model was applied for the network meta-analysis. The relative ranking of interventions was estimated using the surface under the cumulative ranking curve (SUCRA), with a larger SUCRA value indicating a higher probability that the intervention would rank among the best treatment options. Comparison-adjusted funnel plots were used to explore small-study effects and potential publication bias in the network meta-analysis. For multi-arm trials, within-study correlations were taken into account in the analysis. A two-sided *P* value of < 0.05 was considered statistically significant.

## 3 Results

### 3.1 Search results

A total of 2,856 records were identified through database searching. After removal of duplicates using EndNote software, 1,932 records remained for title and abstract screening, of which 1,864 were excluded. The remaining 68 articles were assessed in full text for eligibility. According to the prespecified inclusion and exclusion criteria, 26 randomized controlled trials were ultimately included in the systematic review and network meta-analysis. The study selection process and reasons for full-text exclusion are shown in Figure 1.

**Figure 1:**
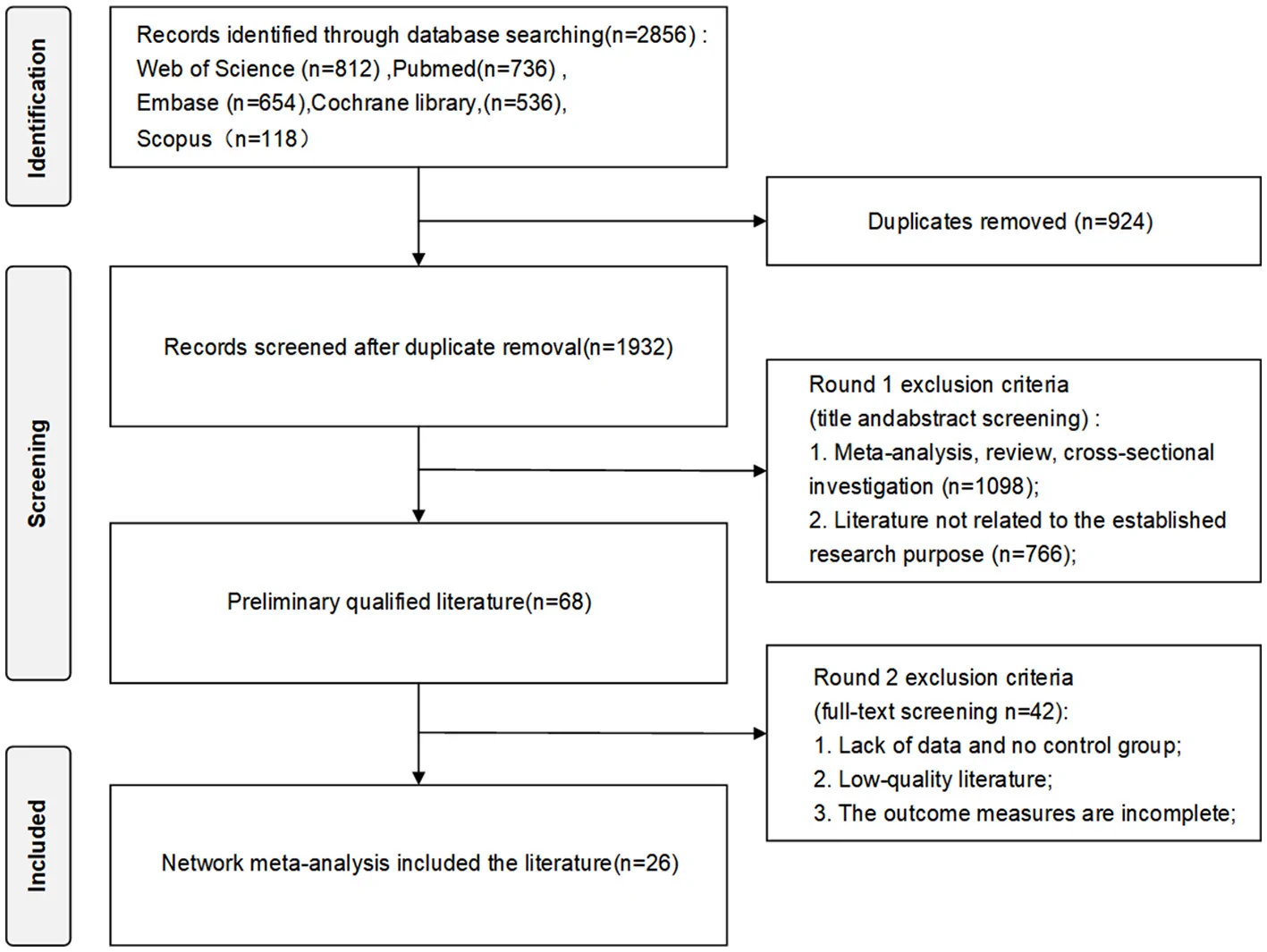
PRISMA flow diagram of the study selection process.

### 3.2 Study characteristics

A total of 26 randomized controlled trials were ultimately included in this study, involving 1,384 patients with adolescent idiopathic scoliosis and 59 study arms. The included studies were published between 2014 and 2025, and were mainly conducted in China, Turkey, Korea, Switzerland, Canada, Italy, Germany, Egypt, Saudi Arabia, and India. The included interventions comprised both single conservative interventions and combined conservative interventions. Single conservative interventions mainly included Schroth exercises, orthotic treatment, proprioceptive neuromuscular facilitation, Pilates, core stabilization exercise, PSSE, Scientific Exercises Approach to Scoliosis (SEAS), home corrective exercise education, and digital care. Combined conservative interventions mainly included Schroth exercises combined with pelvic rotation correction, Schroth exercises combined with balance training, Schroth exercises combined with suspension training, Schroth exercises combined with hippotherapy, and orthotic treatment combined with SEAS.

There was some variation across studies in intervention frequency, session duration, and treatment duration, with most interventions lasting 6 to 24 weeks. The main outcomes included Cobb angle, ATR, and SRS-22 score. Detailed characteristics of the included studies are presented in Table 1.

**Table 1:** Characteristics of randomized controlled trials included in the network meta-analysis.

References	Year	Country	Intervention (E/C)	Sample size (E/C)	Age, year (E/C)	Sex, *n* (M/F), E/C	BMI, kg/m^2^ (E/C)	Baseline Cobb angle, ° (E/C)	Curve location/type	Risser grade (E/C)	Session duration	Freq_week	Period_week	Outcomes
Yuan et al. (34)	2025	China	DC/PSSE	64/64	10.95 ± 2.13/11.31 ± 2.33	15/49 vs. 16/48	16.82 ± 2.14/16.85 ± 2.21	≥10°/≥10°	PUMCH-SSE classification reported	0–4/0–4	30 min	7	24	①②
Yu et al. (35)	2025	China	SE/RC	19/18	9.87 ± 1.23/9.65 ± 1.18	0/19 vs. 0/18	NA	12.15 ± 1.32/11.98 ± 1.25	Mild AIS; left or right curve	NA	60 min	5	8	①②③
Kaya et al. (36)	2025	Turkey	SE/PNF	34/33	13.8 ± 1.6/14.1 ± 1.8	10/24 vs. 8/25	22.3 ± 2.9/21.9 ± 2.3	10–30°/10–30°	Thoracic only; Lenke type 1	0–3/0–3	60 min	3	24	①②③
Zhang et al. (37)	2024	China	PRC + SE/SE	21/21	13.10 ± 2.23/13.57 ± 2.60	6/15 vs. 4/17	18.42 ± 2.72/18.70 ± 2.24	10–25°/10–25°	Mild AIS	0–5/0–5	90 min	1	24	①②③
Zhang et al. (38)	2024	China	SE + ST/SE	31/29	13.42 ± 1.06/13.97 ± 1.21	7/24 vs. 5/24	18.01 ± 1.02/18.07 ± 1.22	<25°/<25°	Lenke type 5; thoracolumbar/lumbar	≤4/≤4	90 min	3	12	①②③
Manzak Dursun et al. (39)	2024	Turkey	PE/RE	16/15	NA	1/15 vs. 0/15	17.72 ± 2.26/19.91 ± 3.41	25–40°/25–40°	NA	NA	60 min	7	12	①②
Shen et al. (26)	2023	China	SE + BE/SE	30/29	13.40 ± 1.01/13.75 ± 1.02	NA	20.09 ± 0.97/20.06 ± 0.81	10–25°/10–25°	Mild AIS	<5/<5	90 min	3	6	①②③
Akyurek et al. (40)	2022	Turkey	PSSE/RE	15/14	13.73 ± 1.83/13.86 ± 1.86	NA	18.75 ± 1.34/19.65 ± 1.84	Thoracic: 19.41 ± 7.03; lumbar: 20.76 ± 7.24/similar baseline	Thoracic and lumbar curves	3–5/3–5	60 min	2	8	②
Wang et al. (41)	2022	China	Insoles + OT/OT	16/15	12.94 ± 1.39/13.67 ± 1.35	3/13 vs. 3/12	18.16 ± 2.05/19.56 ± 1.91	20–45°/20–45°	Moderate AIS; right limb dominance	≤3/≤3	1,380 min	3	24	①②③
Lau et al. (42)	2021	China	HE/RC	20/20	12.8 ± 0.9/13.2 ± 1.1	0/20 vs. 0/20	17.5 ± 5.2/18.5 ± 2.5	15–40°/15–40°	Female AIS only	NA	7 min	5	24	③
Mohamed and Yousef (43)	2021	Egypt	SE/PNF	17/17	14.5 ± 1.2/14.9 ± 1.4	0/17 vs. 0/17	19.25 ± 1.23/19.90 ± 1.60	<25°/<25°	Right single thoracolumbar curve	II–V/II–V	60 min	3	24	①②
Kocaman et al. (44)	2021	Turkey	SE/CSE	14/14	14.07 ± 2.37/14.21 ± 2.19	4/10 vs. 3/11	19.77 ± 4.21/19.73 ± 2.36	10–30°/10–30°	Lenke type 1	≤3/≤3	90 min	3	10	①②③
Dufvenberg et al. (45)^d^	2021	Switzerland	OT/PSSE	45/45	12.7 ± 1.4/12.6 ± 1.4	6/39 vs. 12/33	18.0 ± 2.7/18.2 ± 2.4	25–40°/25–40°	Curve apex T7 or caudal	Growth remaining >1 year; menarche ≤ 1 year	480 min	7	24	①②③
			OT/RE	45/45	12.7 ± 1.4/12.6 ± 1.5	6/39 vs. 6/39	18.0 ± 2.7/18.6 ± 2.7	25–40°/25–40°	Curve apex T7 or caudal	Growth remaining >1 year; menarche ≤ 1 year	60 min	7	23	①②③
Won et al. (46)	2021	Korea	CSE/SEAS	10/10	14.5 ± 2.5/15.9 ± 2.7	0/10 vs. 0/10	NA	>10°/>10°	Left lumbar scoliosis	NA	30 min	3	24	①
Abdel-Aziem et al. (47)	2022	Saudi Arabia	SE + HT/SE	27/25	14.74 ± 1.79/15.04 ± 1.81	8/19 vs. 7/18	19.77 ± 1.02/20.21 ± 1.05	18.59 ± 2.66/19.32 ± 2.69	AIS, all curve types	NA	90 min	3	10	①②
Gao et al. (48)	2019	China	SEAS + OT/OT	23/22	12.22 (10–14)/12.13 (10–14)	NA	NA	25–40°/25–40°	AIS meeting SRS brace criteria	0–2/0–2	40 min + OT 1,380 min	1 + 7	24	①
Yagci et al. (49)	2018	Turkey	SE + OT/SEAS + OT	10/10	14.20 ± 2.04/13.60 ± 1.65	0/10 vs. 0/10	18.93 ± 4.13/18.24 ± 2.57	Thoracic and lumbar Cobb both reported	King types 1–4	1–3/1–3	60 min	2 + 5 home sessions	10	①②③
Zheng et al. (50)	2018	China	SEAS/OT	29/24	12.4 ± 0.9/12.3 ± 0.8	7/22 vs. 5/19	17.6 ± 1.7/18.6 ± 2.5	20–40°/20–40°	Moderate AIS	0–2/0–2	40 min	7	52	①②③
Langensiepen et al. (51)	2017	Germany	SE/RE	20/18	13.6 ± 1.6/14.0 ± 0.9	0/20 vs. 0/18	19.6 ± 3.1/20.2 ± 2.3	Moderate AIS	Girls with brace use ≥16 h/days	NA	12 min	5	24	①
Gür et al. (52)	2017	Turkey	CSE + OT/RE	12/13	14.2 ± 1.8/14.0 ± 1.6	1/11 vs. 0/13	18.0 ± 1.6/18.2 ± 2.3	Brace-referred AIS	King classification recorded	Risser recorded	60 min	7	10	①②③
Kumar et al. (53)	2017	India	PSSE/RE	18/18	12.17 ± 1.72/11.56 ± 1.46	7/11 vs. 8/10	NA	Thoracic < 20° or thoracolumbar < 15°	AIS, fixed deformity	NA	NA	NA	52	①
Schreiber et al. (54)	2016	Canada	PSSE/RC	25/25	13.5 (12.7–14.2)/13.3 (12.7–13.9)	2/23 vs. 1/24	NA	10–45°/10–45°	All curve types	0–5/0–5	30–45 min home + 60 min supervised	7	24	①
Kim and Hwangbo (55)	2016	Korea	SE/PE	12/12	15.6 ± 1.1/15.3 ± 0.8	0/12 vs. 0/12	NA	23.6 ± 1.5/24.0 ± 2.6	Female students with scoliosis	NA	60 min	3	12	①
Kuru et al. (56)	2015	Turkey	SE/RC	15/15	12.9 ± 1.4/12.8 ± 1.2	1/14 vs. 2/13	18.8 ± 3.2/18.0 ± 2.2	10–40°/10–40°	AIS, curve type NA	0–3/0–3	90 min	3 then 7	24	①②③
Wei et al. (57)	2015	China	CMCT/OT	58/49	9.1 ± 0.4/8.9 ± 0.6	18/40 vs. 10/39	NA	20–40°/20–40°	Mild-to-moderate AIS	0–2/0–2	60 min	7	52	①
Monticone et al. (58)	2014	Italy	SE/RE	55/55	12.5 ± 1.1/12.4 ± 1.1	16/39 vs. 14/41	NA	10–25°/10–25°	Thoracic/lumbar/thoracolumbar/S-shaped	<2/<2	60 min	3	184	①②③

BMI, body mass index; NA, not reported; SE, Schroth exercise; PSSE, physiotherapeutic scoliosis-specific exercises; SEAS, Scientific Exercises Approach to Scoliosis; CSE, core stability exercise; PE, Pilates exercise; RE, routine exercise; RC, routine care; LE, Lyon exercise; PNF, proprioceptive neuromuscular facilitation; CMCT, combined traditional Chinese medicine therapy; BE, balance exercise; ST, suspension training; HE, home exercise; HT, hippotherapy; DC, digital care; OT, orthotic treatment; Insoles, customized insoles; PRC, postural response correction. Age and BMI are presented as mean ± standard deviation unless otherwise indicated. Session duration refers to the duration of each treatment session unless otherwise specified. Intervention frequency refers to the number of treatment days or sessions per week as reported in the original studies.

①Cobb angle.

②Angle of trunk rotation (ATR).

③Quality of life score.

^A^Dufvenberg et al. (45) was a three-arm study and is presented as two pairwise comparisons in this table.

### 3.3 Risk of bias and methodological quality

All included studies were randomized controlled trials. Because strict blinding of participants and personnel is difficult to implement in exercise training, home-based training, and brace-related interventions, a certain risk of performance bias may exist in this domain. The assessment of the remaining items, such as random sequence generation, allocation concealment, completeness of outcome data, and selective reporting, still needs to be determined in conjunction with the original risk-of-bias figure. The overall results of the quality assessment are shown in Figure 2.

**Figure 2:**
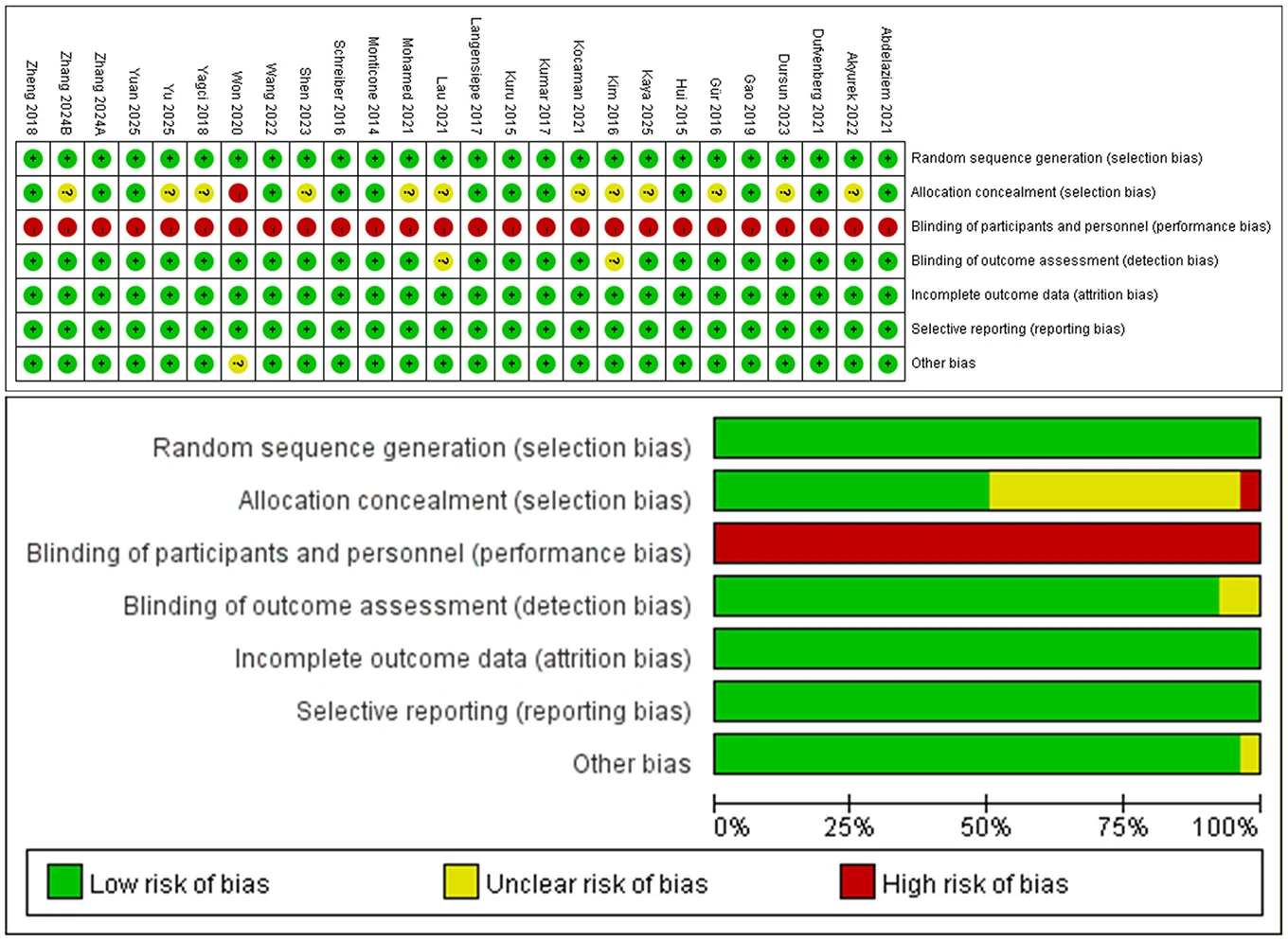
Risk of bias assessment of the included studies.

### 3.4 Network meta-analysis results

#### 3.4.1 Network meta-analysis results for Cobb angle

A total of 24 randomized controlled trials were included for the Cobb angle outcome, involving 15 single and combined conservative interventions. The network plot is shown in Figure 3. In the network graph, each node represents an intervention, and the size of the node reflects the cumulative sample size for that intervention. The lines between nodes indicate direct comparisons, with thicker lines representing a larger number of studies for a given comparison. As shown in Figure 3, Schroth exercise (SE) was the central node in the network and had direct comparisons with routine exercise (RE), orthotic treatment (OT), and several other interventions. In particular, the connections between SE and RE, as well as between SE and OT, were relatively thick, indicating that direct evidence for these comparisons was relatively abundant. The overall network contained multiple closed loops, suggesting that both direct and indirect evidence were available for this outcome and providing the basis for conducting a network meta-analysis.

**Figure 3:**
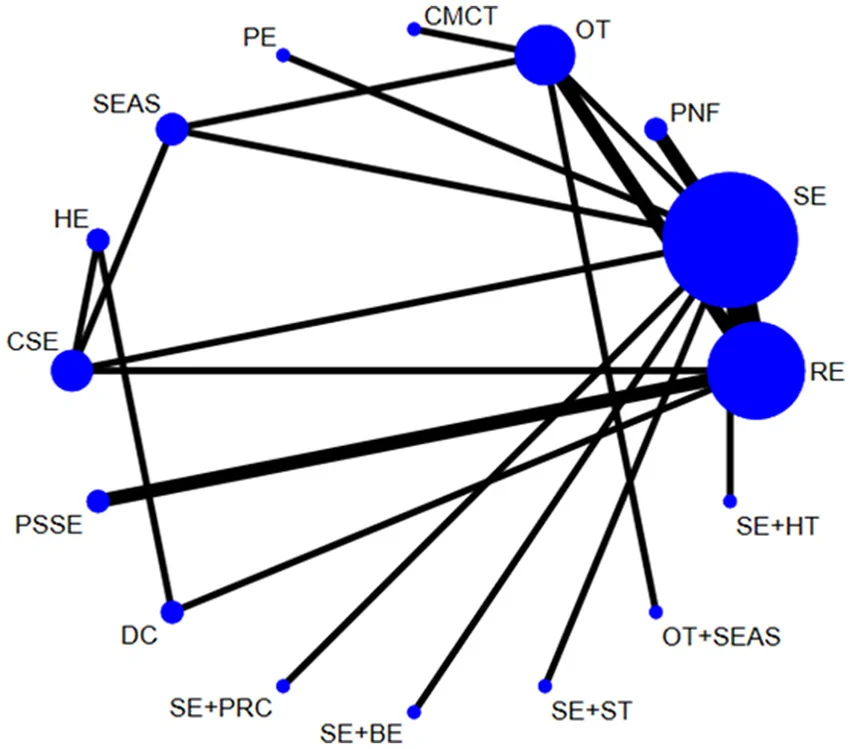
Network plot for Cobb angle. Each node represents an intervention. The size of each node is proportional to the total sample size of that intervention, and the thickness of each connecting line is proportional to the number of studies providing direct comparisons between the two connected interventions. SE, Schroth exercise; PSSE, physiotherapeutic scoliosis-specific exercises; SEAS, Scientific Exercises Approach to Scoliosis; CSE, core stability exercise; PE, Pilates exercise; RE, routine exercise; PNF, proprioceptive neuromuscular facilitation; CMCT, combined traditional Chinese medicine therapy; HE, home exercise; HT, hippotherapy; DC, digital care; OT, orthotic treatment; ST, suspension training; BE, balance exercise; PRC, postural response correction.

The intervention ranking results are shown in Figure 4. SUCRA rankings indicated that Schroth exercise combined with hippotherapy (SE+HT), orthotic treatment combined with Scientific Exercises Approach to Scoliosis (OT+SEAS), and orthotic treatment (OT) ranked relatively high, suggesting that these interventions might have some relative advantages in improving Cobb angle. In contrast, some single exercise-based interventions or combined interventions with relatively small sample sizes ranked lower. It should be noted that SUCRA reflects the relative probability of an intervention being among the better treatment options, rather than directly proving that its efficacy is significantly superior to all other interventions. Therefore, the ranking results should be interpreted in conjunction with the league table.

**Figure 4:**
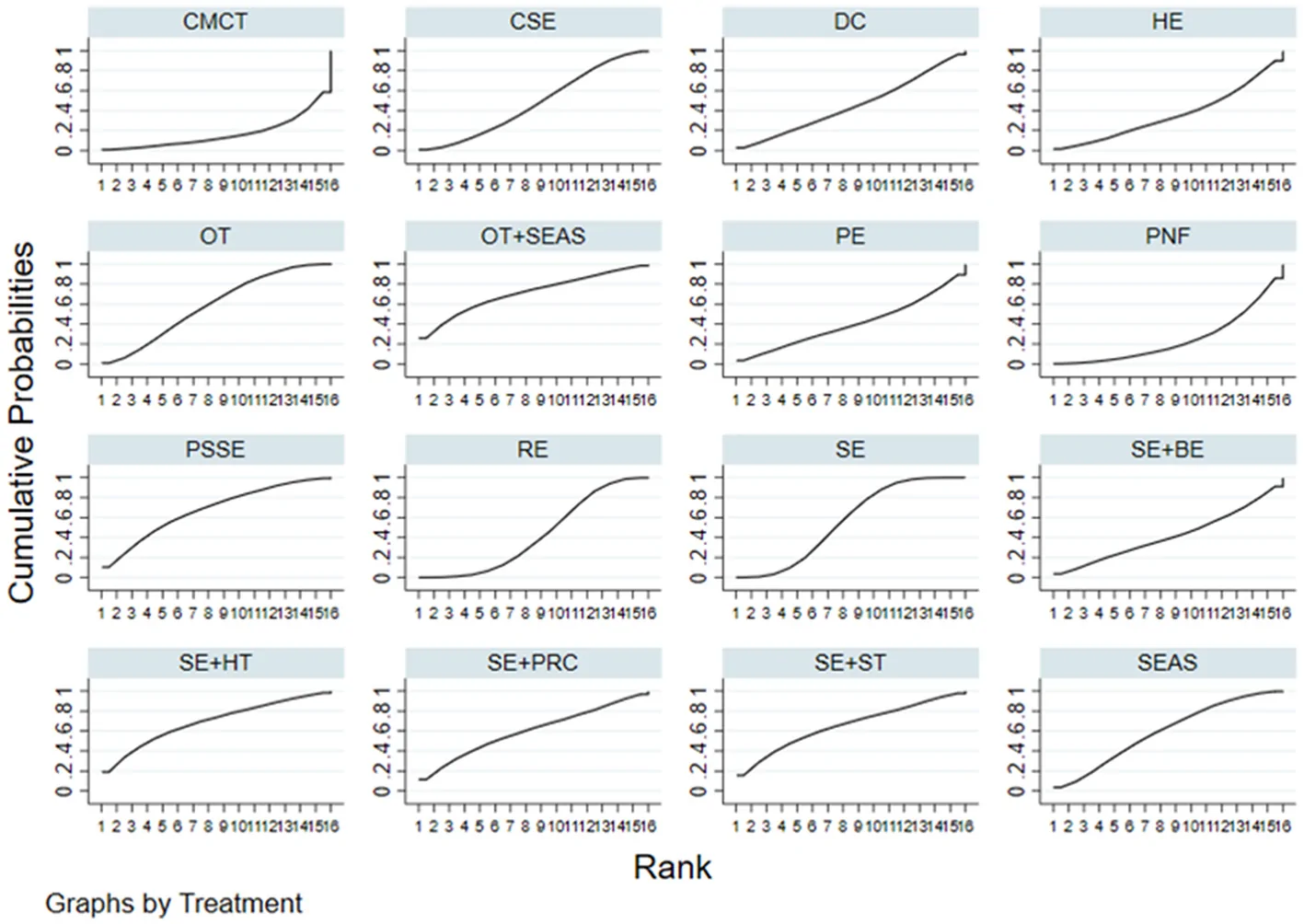
SUCRA ranking plot for Cobb angle. CMCT, combined traditional Chinese medicine therapy; CSE, core stability exercise; DC, digital care; HE, home exercise; OT, orthotic treatment; OT+SEAS, orthotic treatment plus Scientific Exercises Approach to Scoliosis; PE, Pilates exercise; PNF, proprioceptive neuromuscular facilitation; PSSE, physiotherapeutic scoliosis-specific exercises; RE, routine exercise; SE, Schroth exercise; SE+BE, Schroth exercise plus balance exercise; SE+HT, Schroth exercise plus hippotherapy; SE+PRC, Schroth exercise plus postural response correction; SE+ST, Schroth exercise plus suspension training; SEAS, Scientific Exercises Approach to Scoliosis.

The league table results are presented in Table 2. A total of 105 pairwise comparisons among the 15 interventions were generated. Overall, the 95% confidence intervals for most comparisons crossed zero, indicating that no clear statistically significant differences were observed among most interventions based on the available evidence. In particular, for SE+HT, which ranked highly in the SUCRA analysis, the 95% confidence intervals for comparisons with RE, SE, proprioceptive neuromuscular facilitation (PNF), OT, and other single or combined conservative interventions all crossed zero. This suggests that although SE+HT ranked highly, its apparent relative advantage was not consistently supported by pairwise comparisons. Therefore, the ranking shown in Figure 4 should be interpreted as reflecting a relative probability trend rather than as evidence that any one intervention has been definitively proven to be superior.

**Table 2:** League table for Cobb angle.

RE	SE	PNF	OT	CMCT	PE	SEAS	HE	CSE	PSSE	DC	SE+PRC	SE+BE	SE+ST	SE+HT
RE	−0.42 (−1.72, 0.88)	0.85 (−1.57, 3.27)	−0.54 (−2.28, 1.20)	1.65 (−1.68, 4.99)	0.23 (−2.97, 3.44)	−0.56 (−2.60, 1.48)	0.40 (−2.27, 3.07)	−0.14 (−1.95, 1.67)	−0.97 (−3.02, 1.08)	−0.00 (−2.41, 2.41)	−0.70 (−3.86, 2.45)	0.12 (−3.01, 3.26)	−0.97 (−4.11, 2.17)	−1.18 (−4.32, 1.97)
0.42 (−0.88, 1.72)	SE	1.27 (−0.77, 3.31)	−0.12 (−1.96, 1.72)	2.07 (−1.32, 5.46)	0.65 (−2.28, 3.58)	−0.14 (−2.07, 1.79)	0.82 (−1.96, 3.61)	0.29 (−1.54, 2.11)	−0.55 (−2.97, 1.88)	0.42 (−2.22, 3.06)	−0.28 (−3.15, 2.60)	0.54 (−2.31, 3.40)	−0.55 (−3.41, 2.31)	−0.75 (−3.62, 2.11)
−0.85 (−3.27, 1.57)	−1.27 (−3.31, 0.77)	PNF	−1.39 (−4.14, 1.36)	0.81 (−3.15, 4.76)	−0.62 (−4.18, 2.95)	−1.40 (−4.21, 1.40)	−0.44 (−3.90, 3.01)	−0.98 (−3.72, 1.75)	−1.82 (−4.99, 1.35)	−0.85 (−4.18, 2.49)	−1.55 (−5.07, 1.98)	−0.72 (−4.23, 2.78)	−1.82 (−5.33, 1.69)	−2.02 (−5.54, 1.49)
0.54 (−1.20, 2.28)	0.12 (−1.72, 1.96)	1.39 (−1.36, 4.14)	OT	2.19 (−0.65, 5.04)	0.77 (−2.69, 4.23)	−0.02 (−2.08, 2.05)	0.94 (−2.11, 4.00)	0.41 (−1.84, 2.65)	−0.43 (−3.12, 2.26)	0.54 (−2.36, 3.45)	−0.16 (−3.57, 3.26)	0.66 (−2.73, 4.06)	−0.43 (−3.83, 2.97)	−0.64 (−4.04, 2.77)
−1.65 (−4.99, 1.68)	−2.07 (−5.46, 1.32)	−0.81 (−4.76, 3.15)	−2.19 (−5.04, 0.65)	CMCT	−1.42 (−5.90, 3.06)	−2.21 (−5.73, 1.31)	−1.25 (−5.43, 2.93)	−1.79 (−5.41, 1.84)	−2.62 (−6.54, 1.29)	−1.65 (−5.72, 2.41)	−2.35 (−6.80, 2.09)	−1.53 (−5.96, 2.90)	−2.62 (−7.06, 1.81)	−2.83 (−7.27, 1.61)
−0.23 (−3.44, 2.97)	−0.65 (−3.58, 2.28)	0.62 (−2.95, 4.18)	−0.77 (−4.23, 2.69)	1.42 (−3.06, 5.90)	PE	−0.79 (−4.30, 2.72)	0.17 (−3.87, 4.21)	−0.37 (−3.82, 3.08)	−1.20 (−5.00, 2.60)	−0.23 (−4.18, 3.71)	−0.93 (−5.04, 3.17)	−0.11 (−4.20, 3.98)	−1.20 (−5.29, 2.89)	−1.41 (−5.50, 2.69)
0.56 (−1.48, 2.60)	0.14 (−1.79, 2.07)	1.40 (−1.40, 4.21)	0.02 (−2.05, 2.08)	2.21 (−1.31, 5.73)	0.79 (−2.72, 4.30)	SEAS	0.96 (−2.09, 4.01)	0.42 (−1.66, 2.51)	−0.41 (−3.30, 2.48)	0.56 (−2.44, 3.56)	−0.14 (−3.60, 3.32)	0.68 (−2.76, 4.13)	−0.41 (−3.86, 3.03)	−0.62 (−4.07, 2.84)
−0.40 (−3.07, 2.27)	−0.82 (−3.61, 1.96)	0.44 (−3.01, 3.90)	−0.94 (−4.00, 2.11)	1.25 (−2.93, 5.43)	−0.17 (−4.21, 3.87)	−0.96 (−4.01, 2.09)	HE	−0.54 (−3.02, 1.94)	−1.37 (−4.74, 1.99)	−0.40 (−2.85, 2.04)	−1.10 (−5.11, 2.90)	−0.28 (−4.27, 3.71)	−1.37 (−5.36, 2.61)	−1.58 (−5.57, 2.42)
0.14 (−1.67, 1.95)	−0.29 (−2.11, 1.54)	0.98 (−1.75, 3.72)	−0.41 (−2.65, 1.84)	1.79 (−1.84, 5.41)	0.37 (−3.08, 3.82)	−0.42 (−2.51, 1.66)	0.54 (−1.94, 3.02)	CSE	−0.83 (−3.57, 1.90)	0.14 (−2.51, 2.78)	−0.56 (−3.97, 2.84)	0.26 (−3.13, 3.64)	−0.84 (−4.23, 2.55)	−1.04 (−4.44, 2.36)
0.97 (−1.08, 3.02)	0.55 (−1.88, 2.97)	1.82 (−1.35, 4.99)	0.43 (−2.26, 3.12)	2.62 (−1.29, 6.54)	1.20 (−2.60, 5.00)	0.41 (−2.48, 3.30)	1.37 (−1.99, 4.74)	0.83 (−1.90, 3.57)	PSSE	0.97 (−2.19, 4.13)	0.27 (−3.49, 4.03)	1.09 (−2.65, 4.84)	−0.00 (−3.75, 3.74)	−0.21 (−3.96, 3.55)
0.00 (−2.41, 2.41)	−0.42 (−3.06, 2.22)	0.85 (−2.49, 4.18)	−0.54 (−3.45, 2.36)	1.65 (−2.41, 5.72)	0.23 (−3.71, 4.18)	−0.56 (−3.56, 2.44)	0.40 (−2.04, 2.85)	−0.14 (−2.78, 2.51)	−0.97 (−4.13, 2.19)	DC	−0.70 (−4.60, 3.20)	0.12 (−3.77, 4.01)	−0.97 (−4.86, 2.92)	−1.18 (−5.07, 2.72)
0.70 (−2.45, 3.86)	0.28 (−2.60, 3.15)	1.55 (−1.98, 5.07)	0.16 (−3.26, 3.57)	2.35 (−2.09, 6.80)	0.93 (−3.17, 5.04)	0.14 (−3.32, 3.60)	1.10 (−2.90, 5.11)	0.56 (−2.84, 3.97)	−0.27 (−4.03, 3.49)	0.70 (−3.20, 4.60)	SE+PRC	0.82 (−3.23, 4.87)	−0.27 (−4.32, 3.78)	−0.48 (−4.53, 3.58)
−0.12 (−3.26, 3.01)	−0.54 (−3.40, 2.31)	0.72 (−2.78, 4.23)	−0.66 (−4.06, 2.73)	1.53 (−2.90, 5.96)	0.11 (−3.98, 4.20)	−0.68 (−4.13, 2.76)	0.28 (−3.71, 4.27)	−0.26 (−3.64, 3.13)	−1.09 (−4.84, 2.65)	−0.12 (−4.01, 3.77)	−0.82 (−4.87, 3.23)	SE+BE	−1.10 (−5.13, 2.94)	−1.30 (−5.34, 2.75)
0.97 (−2.17, 4.11)	0.55 (−2.31, 3.41)	1.82 (−1.69, 5.33)	0.43 (−2.97, 3.83)	2.62 (−1.81, 7.06)	1.20 (−2.89, 5.29)	0.41 (−3.03, 3.86)	1.37 (−2.61, 5.36)	0.84 (−2.55, 4.23)	0.00 (−3.74, 3.75)	0.97 (−2.92, 4.86)	0.27 (−3.78, 4.32)	1.10 (−2.94, 5.13)	SE+ST	−0.20 (−4.25, 3.84)
1.34 (−2.02, 4.70)	0.92 (−2.50, 4.33)	2.19 (−1.79, 6.16)	0.80 (−2.08, 3.67)	2.99 (−1.05, 7.04)	1.57 (−2.93, 6.07)	0.78 (−2.76, 4.32)	1.74 (−2.46, 5.94)	1.20 (−2.45, 4.85)	0.37 (−3.57, 4.31)	1.34 (−2.75, 5.43)	0.64 (−3.82, 5.10)	1.46 (−2.99, 5.91)	0.37 (−4.09, 4.82)	0.16 (−4.29, 4.62)
1.18 (−1.97, 4.32)	0.75 (−2.11, 3.62)	2.02 (−1.49, 5.54)	0.64 (−2.77, 4.04)	2.83 (−1.61, 7.27)	1.41 (−2.69, 5.50)	0.62 (−2.84, 4.07)	1.58 (−2.42, 5.57)	1.04 (−2.36, 4.44)	0.21 (−3.55, 3.96)	1.18 (−2.72, 5.07)	0.48 (−3.58, 4.53)	1.30 (−2.75, 5.34)	0.20 (−3.84, 4.25)	SE+HT

Data are presented as mean difference (95% confidence interval). RE, routine exercise; SE, Schroth exercise; PNF, proprioceptive neuromuscular facilitation; OT, orthotic treatment; CMCT, combined traditional Chinese medicine therapy; PE, Pilates exercise; SEAS, Scientific Exercises Approach to Scoliosis; HE, home exercise; CSE, core stability exercise; PSSE, physiotherapeutic scoliosis-specific exercises; DC, digital care; SE+PRC, Schroth exercise plus postural response correction; SE+BE, Schroth exercise plus balance exercise; SE+ST, Schroth exercise plus suspension training; SE+HT, Schroth exercise plus hippotherapy.

The exploration of publication bias is shown in Figure 5. The comparison-adjusted funnel plot demonstrated that most study points were clustered around the comparison-specific pooled effect size, and the overall distribution was generally within the funnel region. However, some asymmetry was observed between the left and right sides of the plot, and a few outlying points were present, suggesting the possibility of small-study effects or potential publication bias for this outcome.

**Figure 5:**
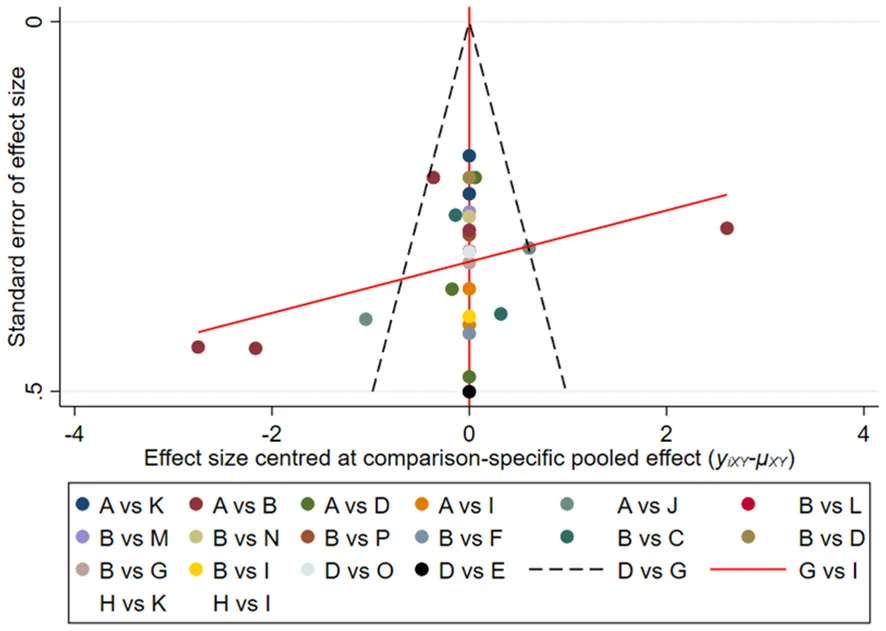
Comparison-adjusted funnel plot for Cobb angle.

#### 3.4.2 Network meta-analysis results for angle of trunk rotation

A total of 16 studies were included for the angle of trunk rotation outcome, involving 11 interventions. The network plot is shown in Figure 6. As illustrated, Schroth exercise (SE) and routine exercise (RE) remained the major central nodes for this outcome and, together with orthotic treatment (OT), Scientific Exercises Approach to Scoliosis (SEAS), core stability exercise (CSE), home exercise (HE), digital care (DC), Schroth exercise combined with pelvic rotation correction (SE+PRC), Schroth exercise combined with balance training (SE+BE), and Schroth exercise combined with suspension training (SE+ST), constituted the main comparison network. Overall, the network was relatively well-connected; however, compared with the Cobb angle outcome, it was smaller in scale and contained fewer closed loops, suggesting that the evidence base for this outcome was relatively limited.

**Figure 6:**
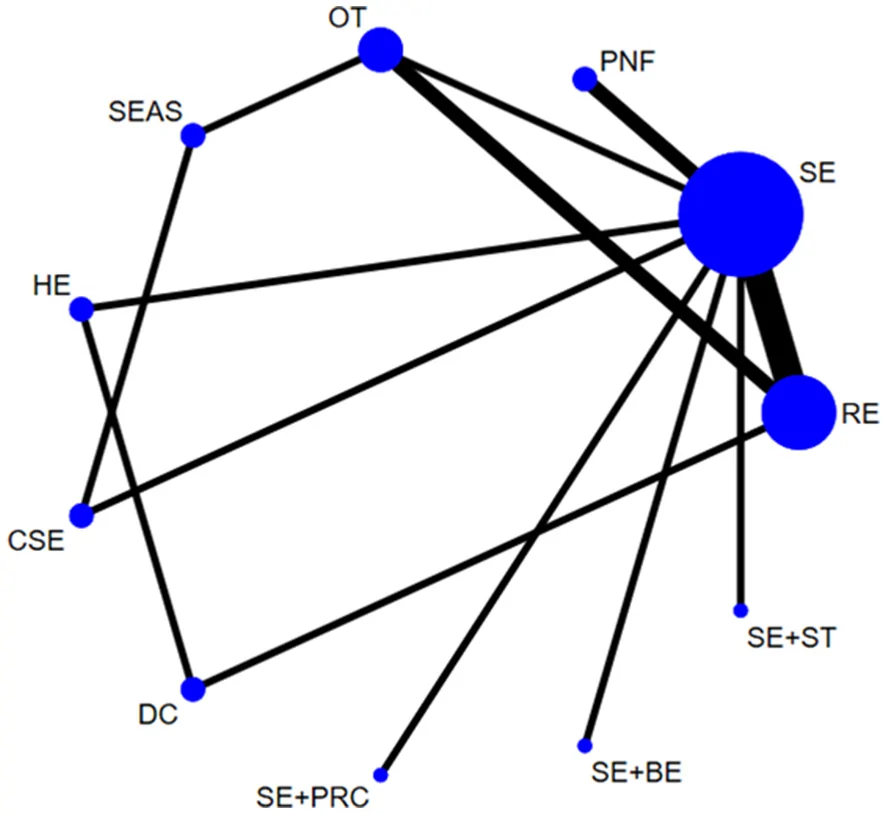
Network plot for ATR. Each node represents an intervention. The size of each node is proportional to the total sample size of that intervention, and the thickness of each connecting line is proportional to the number of studies providing direct comparisons between the two connected interventions. OT, orthotic treatment; PNF, proprioceptive neuromuscular facilitation; SE, Schroth exercise; RE, routine exercise; SE+ST, Schroth exercise plus suspension training; SE+BE, Schroth exercise plus balance exercise; SE+PRC, Schroth exercise plus postural response correction; DC, digital care; CSE, core stability exercise; HE, home exercise; SEAS, Scientific Exercises Approach to Scoliosis.

The intervention ranking results are shown in Figure 7. SUCRA rankings indicated that Schroth exercise combined with suspension training (SE+ST), Schroth exercise (SE), routine exercise (RE), and orthotic treatment (OT) ranked relatively high, suggesting that these interventions might have some relative advantages in improving angle of trunk rotation. In contrast, proprioceptive neuromuscular facilitation (PNF), digital care (DC), and some other interventions ranked relatively lower. As with the Cobb angle outcome, these rankings primarily reflect relative probabilities of treatment performance and should be interpreted together with the league table to determine the extent of statistical support.

**Figure 7:**
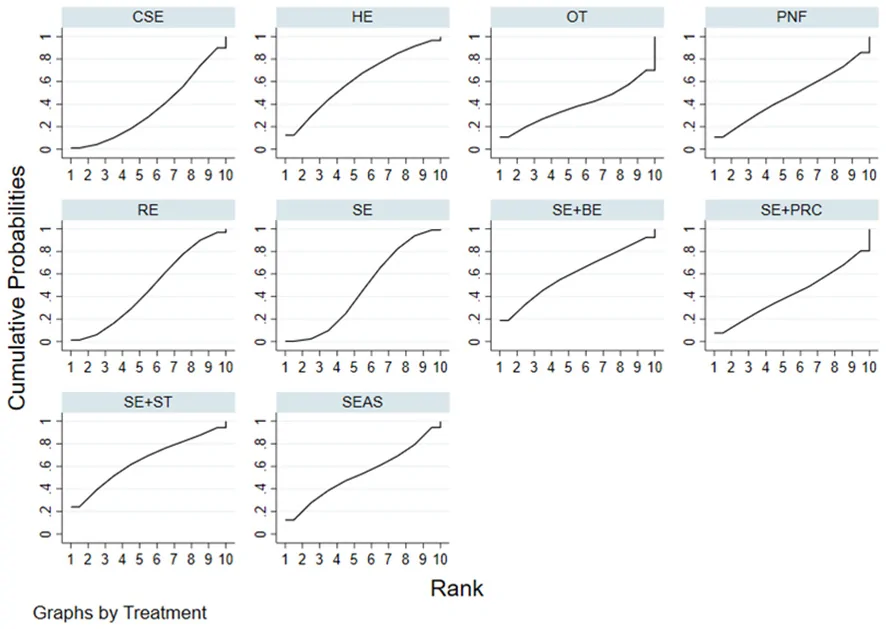
SUCRA ranking plot for ATR. CSE, core stability exercise; DC, digital care; HE, home exercise; OT, orthotic treatment; PNF, proprioceptive neuromuscular facilitation; RE, routine exercise; SE, Schroth exercise; SE+BE, Schroth exercise plus balance exercise; SE+PRC, Schroth exercise plus postural response correction; SE+ST, Schroth exercise plus suspension training; SEAS, Scientific Exercises Approach to Scoliosis.

The league table results are presented in Table 3. A total of 55 pairwise comparisons were generated. Overall, the 95% confidence intervals for most comparisons crossed zero, indicating that no clear statistically significant differences were observed for most intervention comparisons. Only the comparison between Schroth exercise (SE) and proprioceptive neuromuscular facilitation (PNF) reached statistical significance [WMD = 2.94, 95% CI (0.48, 5.39)], suggesting that, based on the combined evidence within this network, SE might have an advantage over PNF in improving angle of trunk rotation. Apart from this comparison, although several higher-ranked interventions occupied favorable positions in the SUCRA plot, there was still insufficient pairwise comparative evidence to support a clear superiority claim.

**Table 3:** League table for ATR.

RE	SE	PNF	OT	SEAS	HE	CSE	DC	SE+PRC	SE+BE	SE+ST
RE	−0.28 (−1.85, 1.29)	2.66 (−0.25, 5.57)	−0.45 (−2.59, 1.69)	−0.62 (−3.84, 2.60)	0.23 (−2.72, 3.18)	0.57 (−2.55, 3.68)	0.23 (−2.55, 3.01)	0.28 (−3.43, 3.99)	−0.83 (−4.53, 2.87)	−1.31 (−5.01, 2.39)
0.28 (−1.29, 1.85)	SE	2.94 (0.48, 5.39)	−0.18 (−2.46, 2.10)	−0.34 (−3.50, 2.81)	0.51 (−2.30, 3.32)	0.84 (−2.03, 3.72)	0.51 (−2.43, 3.44)	0.56 (−2.81, 3.92)	−0.55 (−3.90, 2.80)	−1.03 (−4.38, 2.32)
−2.66 (−5.57, 0.25)	−2.94 (−5.39, −0.48)	PNF	−3.11 (−6.46, 0.24)	−3.28 (−7.28, 0.72)	−2.43 (−6.16, 1.30)	−2.09 (−5.87, 1.69)	−2.43 (−6.25, 1.39)	−2.38 (−6.54, 1.79)	−3.49 (−7.64, 0.67)	−3.97 (−8.12, 0.19)
0.45 (−1.69, 2.59)	0.18 (−2.10, 2.46)	3.11 (−0.24, 6.46)	OT	−0.17 (−3.02, 2.69)	0.68 (−2.79, 4.16)	1.02 (−2.14, 4.18)	0.68 (−2.74, 4.10)	0.73 (−3.33, 4.80)	−0.37 (−4.42, 3.68)	−0.85 (−4.91, 3.20)
0.62 (−2.60, 3.84)	0.34 (−2.81, 3.50)	3.28 (−0.72, 7.28)	0.17 (−2.69, 3.02)	SEAS	0.85 (−3.29, 4.99)	1.19 (−1.72, 4.09)	0.85 (−3.29, 4.99)	0.90 (−3.71, 5.51)	−0.21 (−4.81, 4.40)	−0.69 (−5.29, 3.92)
−0.23 (−3.18, 2.72)	−0.51 (−3.32, 2.30)	2.43 (−1.30, 6.16)	−0.68 (−4.16, 2.79)	−0.85 (−4.99, 3.29)	HE	0.34 (−3.64, 4.32)	−0.00 (−2.81, 2.80)	0.05 (−4.34, 4.43)	−1.06 (−5.43, 3.31)	−1.54 (−5.91, 2.84)
−0.57 (−3.68, 2.55)	−0.84 (−3.72, 2.03)	2.09 (−1.69, 5.87)	−1.02 (−4.18, 2.14)	−1.19 (−4.09, 1.72)	−0.34 (−4.32, 3.64)	CSE	−0.34 (−4.36, 3.68)	−0.29 (−4.71, 4.14)	−1.39 (−5.81, 3.02)	−1.87 (−6.29, 2.54)
−0.23 (−3.01, 2.55)	−0.51 (−3.44, 2.43)	2.43 (−1.39, 6.25)	−0.68 (−4.10, 2.74)	−0.85 (−4.99, 3.29)	0.00 (−2.80, 2.81)	0.34 (−3.68, 4.36)	DC	0.05 (−4.41, 4.51)	−1.06 (−5.51, 3.39)	−1.54 (−5.99, 2.92)
−0.28 (−3.99, 3.43)	−0.56 (−3.92, 2.81)	2.38 (−1.79, 6.54)	−0.73 (−4.80, 3.33)	−0.90 (−5.51, 3.71)	−0.05 (−4.43, 4.34)	0.29 (−4.14, 4.71)	−0.05 (−4.51, 4.41)	SE+PRC	−1.11 (−5.85, 3.64)	−1.59 (−6.34, 3.16)
0.83 (−2.87, 4.53)	0.55 (−2.80, 3.90)	3.49 (−0.67, 7.64)	0.37 (−3.68, 4.42)	0.21 (−4.40, 4.81)	1.06 (−3.31, 5.43)	1.39 (−3.02, 5.81)	1.06 (−3.39, 5.51)	1.11 (−3.64, 5.85)	SE+BE	−0.48 (−5.22, 4.26)
1.31 (−2.39, 5.01)	1.03 (−2.32, 4.38)	3.97 (−0.19, 8.12)	0.85 (−3.20, 4.91)	0.69 (−3.92, 5.29)	1.54 (−2.84, 5.91)	1.87 (−2.54, 6.29)	1.54 (−2.92, 5.99)	1.59 (−3.16, 6.34)	0.48 (−4.26, 5.22)	SE+ST

Data are presented as mean difference (95% confidence interval). RE, routine exercise; SE, Schroth exercise; PNF, proprioceptive neuromuscular facilitation; OT, orthotic treatment; SEAS, Scientific Exercises Approach to Scoliosis; HE, home exercise; CSE, core stability exercise; DC, digital care; SE+PRC, Schroth exercise plus postural response correction; SE+BE, Schroth exercise plus balance exercise; SE+ST, Schroth exercise plus suspension training.

The exploration of publication bias is shown in Figure 8. The comparison-adjusted funnel plot demonstrated that most study points were distributed within the funnel region and clustered around the center line, although some asymmetry was observed between the two sides of the plot, suggesting the possibility of small-study effects or potential publication bias for this outcome.

**Figure 8:**
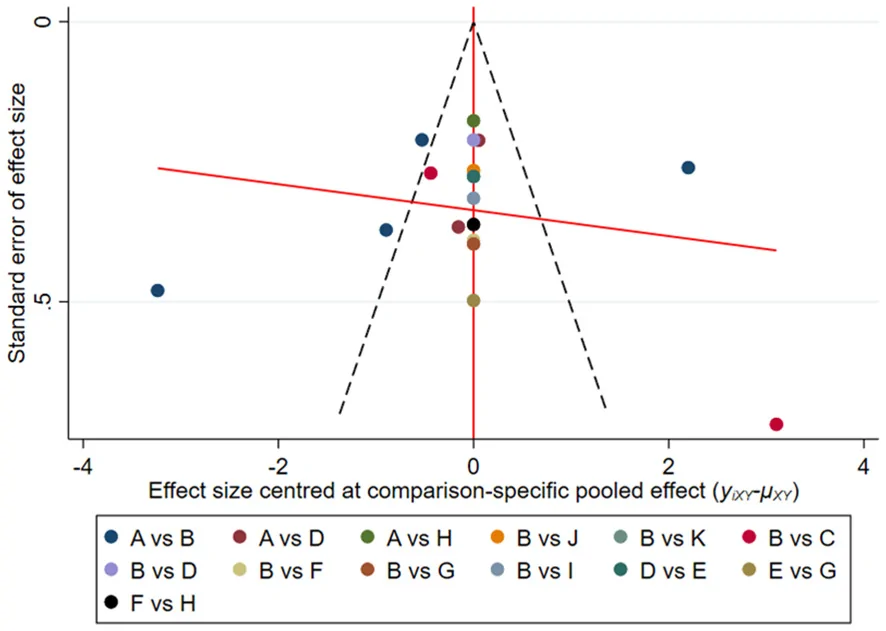
Comparison-adjusted funnel plot for ATR.

#### 3.4.3 Network meta-analysis results for quality of life

Quality of life was assessed using the SRS-22 score. A total of 13 studies were included, involving 10 interventions. The network plot is shown in Figure 9. As illustrated, Schroth exercise (SE) was the major intervention node and had direct comparisons with routine exercise (RE), proprioceptive neuromuscular facilitation (PNF), home exercise (HE), core stability exercise (CSE), and several combined interventions. Among these, the comparison between SE and RE was relatively well-represented. The presence of closed loops in the network suggested that both direct and indirect evidence could be integrated for analysis.

**Figure 9:**
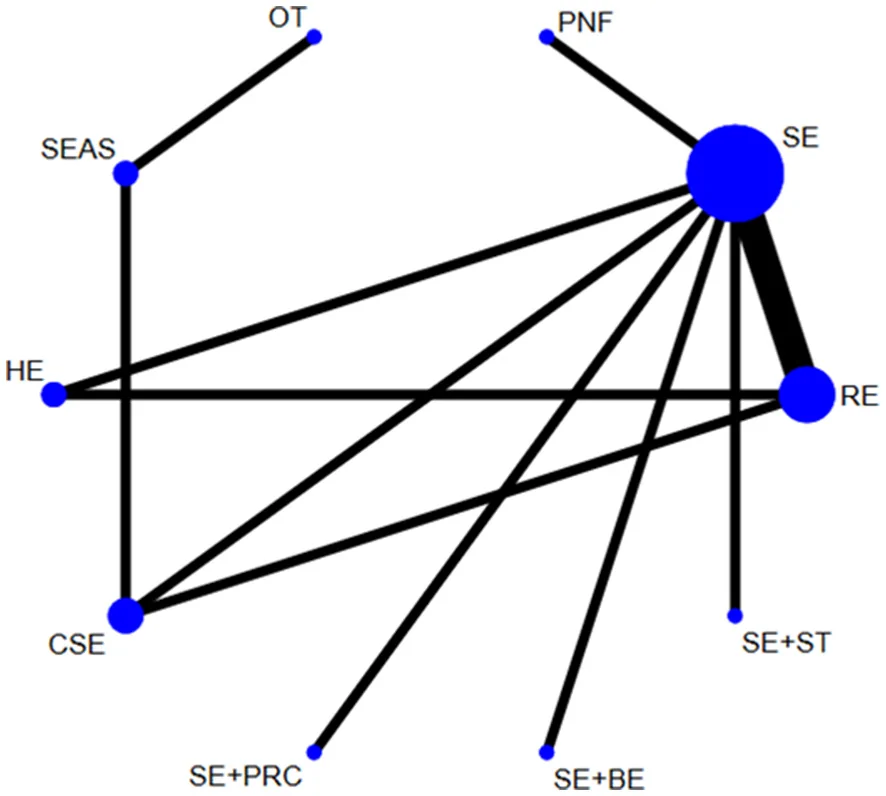
Network plot for quality of life. Each node represents an intervention. The size of each node is proportional to the total sample size of that intervention, and the thickness of each connecting line is proportional to the number of studies providing direct comparisons between the two connected interventions. OT, orthotic treatment; PNF, proprioceptive neuromuscular facilitation; SE, Schroth exercise; RE, routine exercise; SE+ST, Schroth exercise plus suspension training; SE+BE, Schroth exercise plus balance exercise; SE+PRC, Schroth exercise plus postural response correction; CSE, core stability exercise; HE, home exercise; SEAS, Scientific Exercises Approach to Scoliosis.

The intervention ranking results are shown in Figure 10. SUCRA rankings indicated that Schroth exercise combined with suspension training (SE+ST), Schroth exercise (SE), and routine exercise (RE) ranked relatively high, suggesting that these interventions might have some relative advantages in improving SRS-22 scores. In contrast, Schroth exercise combined with pelvic rotation correction (SE+PRC), proprioceptive neuromuscular facilitation (PNF), and core stability exercise (CSE) ranked relatively lower. It should be noted that these results only reflect differences in the ranking probabilities of the interventions and should not be directly interpreted as definitive evidence of superiority or inferiority.

**Figure 10:**
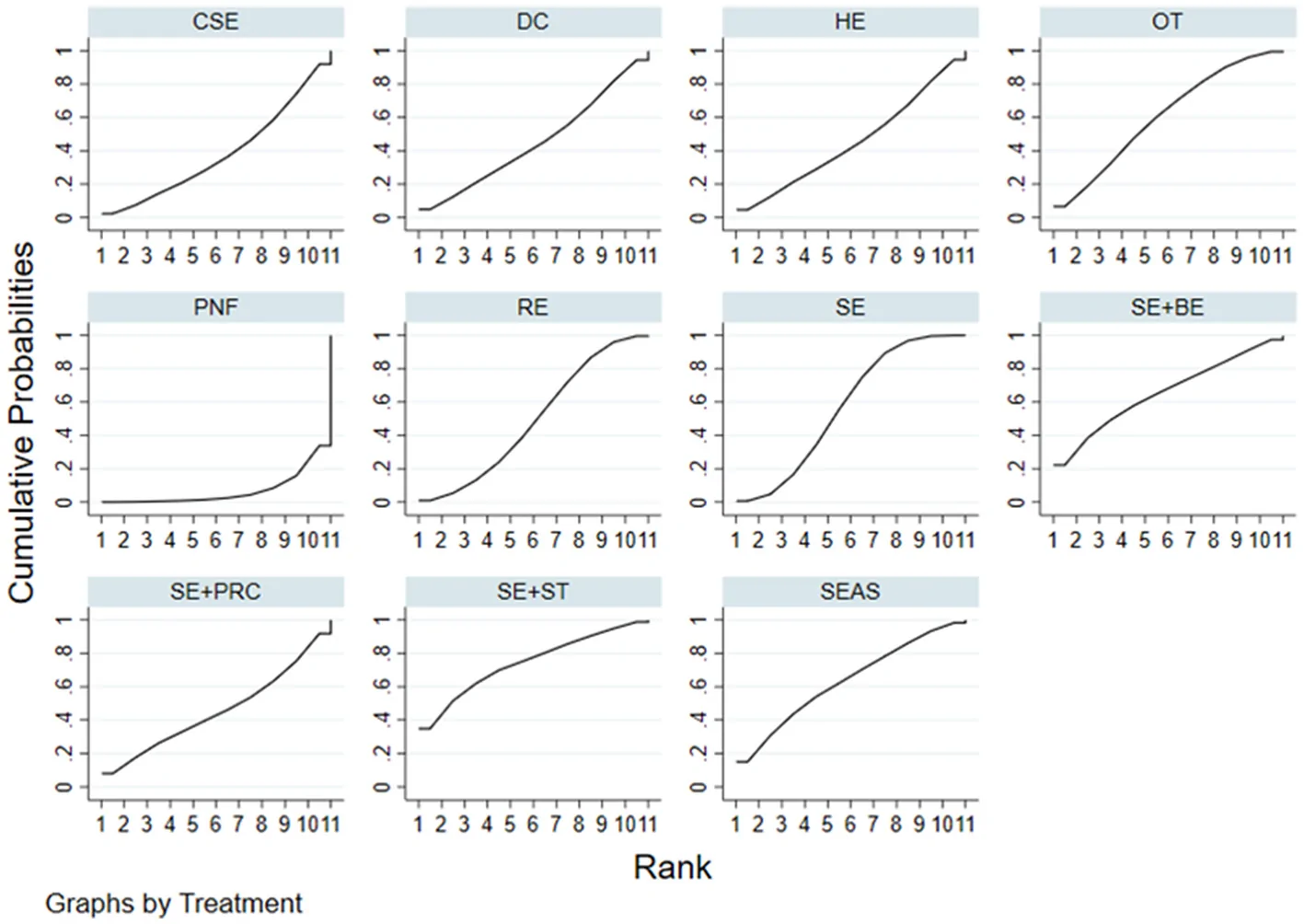
SUCRA ranking plot for quality of life. CSE, core stability exercise; HE, home exercise; OT, orthotic treatment; PNF, proprioceptive neuromuscular facilitation; RE, routine exercise; SE, Schroth exercise; SE+BE, Schroth exercise plus balance exercise; SE+PRC, Schroth exercise plus postural response correction; SE+ST, Schroth exercise plus suspension training; SEAS, Scientific Exercises Approach to Scoliosis.

The league table results are presented in Table 4. A total of 45 pairwise comparisons were generated. The results showed that the 95% confidence intervals for all comparisons crossed zero, indicating that no clear statistically significant differences were observed among the interventions. This suggests that although SE+ST, SE, and RE ranked relatively high in the SUCRA analysis, the current evidence is insufficient to support clear superiority of any intervention in improving quality of life. Therefore, the ranking results for this outcome are more appropriately interpreted as indicating a possible trend toward relative advantage rather than as evidence warranting strong inference.

**Table 4:** League table for quality of life.

RE	SE	PNF	OT	SEAS	HE	CSE	SE+PRC	SE+BE	SE+ST
RE	0.03 (−1.58, 1.64)	0.03 (−3.54, 3.60)	−0.59 (−5.78, 4.60)	0.21 (−3.88, 4.30)	0.63 (−1.78, 3.04)	−0.46 (−2.90, 1.98)	−0.27 (−3.86, 3.32)	0.66 (−2.91, 4.24)	0.90 (−2.68, 4.48)
−0.03 (−1.64, 1.58)	SE	−0.00 (−3.19, 3.19)	−0.62 (−5.82, 4.57)	0.18 (−3.91, 4.27)	0.60 (−1.81, 3.01)	−0.49 (−2.93, 1.96)	−0.30 (−3.51, 2.91)	0.63 (−2.56, 3.83)	0.87 (−2.33, 4.06)
−0.03 (−3.60, 3.54)	0.00 (−3.19, 3.19)	PNF	−0.62 (−6.72, 5.47)	0.18 (−5.01, 5.36)	0.60 (−3.40, 4.60)	−0.49 (−4.51, 3.53)	−0.30 (−4.82, 4.22)	0.63 (−3.88, 5.14)	0.87 (−3.65, 5.38)
0.59 (−4.60, 5.78)	0.62 (−4.57, 5.82)	0.62 (−5.47, 6.72)	OT	0.80 (−2.40, 4.00)	1.22 (−4.39, 6.83)	0.13 (−4.45, 4.71)	0.32 (−5.78, 6.43)	1.25 (−4.84, 7.35)	1.49 (−4.61, 7.59)
−0.21 (−4.30, 3.88)	−0.18 (−4.27, 3.91)	−0.18 (−5.36, 5.01)	−0.80 (−4.00, 2.40)	SEAS	0.42 (−4.18, 5.03)	−0.67 (−3.94, 2.61)	−0.47 (−5.67, 4.72)	0.46 (−4.73, 5.64)	0.69 (−4.50, 5.88)
−0.63 (−3.04, 1.78)	−0.60 (−3.01, 1.81)	−0.60 (−4.60, 3.40)	−1.22 (−6.83, 4.39)	−0.42 (−5.03, 4.18)	HE	−1.09 (−4.33, 2.15)	−0.90 (−4.91, 3.12)	0.03 (−3.97, 4.03)	0.27 (−3.73, 4.27)
0.46 (−1.98, 2.90)	0.49 (−1.96, 2.93)	0.49 (−3.53, 4.51)	−0.13 (−4.71, 4.45)	0.67 (−2.61, 3.94)	1.09 (−2.15, 4.33)	CSE	0.19 (−3.84, 4.23)	1.12 (−2.90, 5.14)	1.36 (−2.67, 5.38)
0.27 (−3.32, 3.86)	0.30 (−2.91, 3.51)	0.30 (−4.22, 4.82)	−0.32 (−6.43, 5.78)	0.47 (−4.72, 5.67)	0.90 (−3.12, 4.91)	−0.19 (−4.23, 3.84)	SE+PRC	0.93 (−3.60, 5.46)	1.17 (−3.36, 5.69)
−0.66 (−4.24, 2.91)	−0.63 (−3.83, 2.56)	−0.63 (−5.14, 3.88)	−1.25 (−7.35, 4.84)	−0.46 (−5.64, 4.73)	−0.03 (−4.03, 3.97)	−1.12 (−5.14, 2.90)	−0.93 (−5.46, 3.60)	SE+BE	0.24 (−4.28, 4.75)
−0.90 (−4.48, 2.68)	−0.87 (−4.06, 2.33)	−0.87 (−5.38, 3.65)	−1.49 (−7.59, 4.61)	−0.69 (−5.88, 4.50)	−0.27 (−4.27, 3.73)	−1.36 (−5.38, 2.67)	−1.17 (−5.69, 3.36)	−0.24 (−4.75, 4.28)	SE+ST

Data are presented as mean difference (95% confidence interval). RE, routine exercise; SE, Schroth exercise; PNF, proprioceptive neuromuscular facilitation; OT, orthotic treatment; SEAS, Scientific Exercises Approach to Scoliosis; HE, home exercise; CSE, core stability exercise; SE+PRC, Schroth exercise plus postural response correction; SE+BE, Schroth exercise plus balance exercise; SE+ST, Schroth exercise plus suspension training.

The exploration of publication bias is shown in Figure 11. The comparison-adjusted funnel plot demonstrated that most studies were distributed within the funnel region and clustered around the center line, although some asymmetry was observed between the two sides of the plot, suggesting the possibility of small-study effects or potential publication bias.

**Figure 11:**
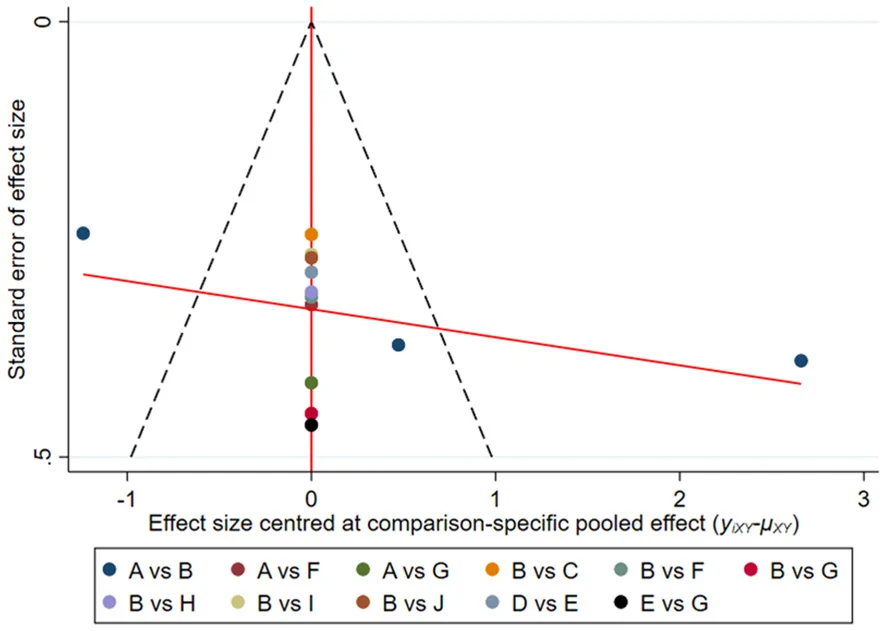
Comparison-adjusted funnel plot for quality of life.

### 3.5 GRADE assessment of the quality of evidence

The GRADE assessment of evidence quality showed that, for the Cobb angle outcome, 24 randomized controlled trials were included. The risk of bias was low, but some inconsistency was present; therefore, the overall quality of evidence was rated as moderate. For angle of trunk rotation, 16 randomized controlled trials were included. Although the risk of bias was low, the overall quality of evidence was rated as low because of substantial inconsistency across studies. For SRS-22, 13 randomized controlled trials were included. The risk of bias was low, with no serious concerns regarding inconsistency or indirectness; however, some imprecision was identified, and thus the overall quality of evidence was rated as moderate. The GRADE assessment results are summarized in Table 5.

**Table 5:** GRADE evaluation of evidence quality across outcomes.

Index	Quality assessment						SDM 95%CI	Confidence rating
	Number of studies	Risk of bias	Inconsistency	Indirectness	Imprecision	Other factors		
Cobb angle	24 RCTs	Low risk	Some concerns	No concerns	No concerns	None	0.25 [−0.10, 0.60]	Moderate
ATR	16 RCTs	Low risk	Major concerns	No concerns	No concerns	None	0.30 [−0.15, 0.75]	Low
SRS-22	13 RCTs	Low risk	No concerns	No concerns	Some concerns	None	0.10 [−0.20, 0.40]	Moderate

The certainty of evidence was assessed using the grading of recommendations assessment, development and evaluation (GRADE) approach. For randomized controlled trials (RCTs), the initial certainty rating was set as high. The rating was downgraded by one level for some concerns or by two levels for major concerns in any of the following domains: risk of bias, inconsistency, indirectness, imprecision, and other factors (e.g., publication bias). High certainty: we are very confident that the true effect lies close to that of the estimate of the effect. Moderate certainty: we are moderately confident in the effect estimate; the true effect is likely to be close to the estimate of the effect, but there is a possibility that it is substantially different. Low certainty: our confidence in the effect estimate is limited; the true effect may be substantially different from the estimate of the effect. Very low certainty: we have very little confidence in the effect estimate; the true effect is likely to be substantially different from the estimate of the effect.

## 4 Discussion

AIS is a three-dimensional spinal deformity that occurs during the growth and developmental period. If not treated in a timely manner, the curvature may continue to progress during the rapid skeletal growth of adolescence, further affecting trunk appearance, postural balance, motor function, and mental health (22–25). Therefore, before surgical indications are reached, selecting a scientific and effective conservative treatment is of important clinical significance. In recent years, non-surgical treatments for AIS have become increasingly diverse, including not only single interventions such as Schroth exercises, orthotic treatment, proprioceptive neuromuscular facilitation, Pilates, core stability training, SEAS scoliosis-specific exercises, home-based corrective exercise education, and digital therapeutics, but also combined interventions such as Schroth exercises combined with balance training, sling training, pelvic rotation correction, hippotherapy, and orthotic treatment combined with SEAS (26). However, previous studies have mostly been limited to pairwise comparisons between a single intervention and conventional treatment, and there is still a lack of systematic evaluation of the relative efficacy of different single and combined conservative interventions. Based on this, the present study conducted a network meta-analysis, including 26 randomized controlled trials and 1,384 AIS patients. After data extraction, 21 interventions were identified. The results showed that, for the outcomes of Cobb angle and angle of trunk rotation, some combined conservative interventions ranked among the top in probability ranking, and combination protocols centered on Schroth exercises demonstrated potential advantages across multiple outcomes. In terms of improvement in SRS-22 scores, some Schroth-based combined interventions also showed a tendency to rank relatively high. However, most pairwise comparisons did not reach statistical significance, suggesting that the current evidence reflects potential advantages rather than definitive superiority. This indicates that, in theory, combined conservative interventions may be more consistent with the clinical characteristics of AIS, in which three-dimensional deformity coexists with functional impairment, but their relative advantages still require further confirmation through more high-quality studies.

The Cobb angle is the core indicator for evaluating the severity of structural scoliosis in AIS and is also the most commonly used index in clinical diagnosis, disease stratification, treatment decision-making, and follow-up assessment of therapeutic effects (27). This study showed that, for the Cobb angle outcome, Schroth exercises combined with hippotherapy, orthotic treatment combined with SEAS scoliosis-specific exercises, and orthotic treatment ranked relatively high in SUCRA, suggesting that these interventions may have better relative efficacy in reducing the Cobb angle. Overall, some combined interventions ranked relatively high for the Cobb angle outcome, suggesting that structural correction in AIS may benefit from the combined effects of active three-dimensional correction, core stability control, dynamic balance training, and external support (28); however, because most pairwise comparisons did not show statistically significant differences, these inferences should be interpreted cautiously. Schroth exercises were one of the core nodes in the evidence network of this study, and their combined protocols performed well across multiple outcomes. This may be related to their emphasis on three-dimensional auto-correction of the spine, rotational angular breathing, and postural awareness training, which can simultaneously improve coronal plane deviation, transverse plane rotation, and sagittal plane abnormalities, while enhancing the coordination and stability of the paraspinal muscle groups. However, given that statistical differences were not stable, the above mechanistic explanation remains largely theoretical.

Among these, Schroth exercises combined with hippotherapy ranked relatively high in this study, which may suggest a potential benefit of combining active three-dimensional correction with dynamic sensory input. However, this finding should be interpreted with considerable caution, because the evidence for this intervention was derived from a very limited number of studies with small sample sizes. Under such circumstances, SUCRA-based rankings may be unstable and may overestimate the apparent advantage of an intervention. Therefore, the favorable ranking of Schroth combined with hippotherapy should be regarded as hypothesis-generating rather than as robust evidence of superiority. Through continuous and rhythmic three-dimensional pelvic movement stimulation, hippotherapy enables patients to continuously adjust the spatial relationship among the pelvis, trunk, and spine under dynamic conditions, thereby improving postural control and trunk balance (29–31). Meanwhile, the high ranking of orthotic treatment combined with SEAS also suggests that the “external support + active exercise” model has good clinical application value (13, 32). Orthotic treatment provides sustained mechanical correction to help control curve progression, whereas exercise improves active self-correction, trunk stability, and postural control. Their combination may therefore integrate structural support with functional retraining, which is particularly relevant for AIS as a condition involving both structural deformity and functional impairment. It should be noted that although the SUCRA ranking suggests a superior trend for some combined interventions, the league table results showed that the 95% confidence intervals of most pairwise comparisons crossed 0, and no stable or definitive statistical differences have yet been established. Therefore, the Cobb angle outcome currently reflects relative efficacy ranking rather than absolute superiority of any one intervention over all others.

In addition to the Cobb angle, the angle of trunk rotation is also an important indicator for evaluating AIS treatment efficacy, as it more directly reflects surface rotational deformity and trunk asymmetry and is closely associated with postural stability and appearance perception. A total of 16 studies were included in the analysis of this outcome. The results showed that Schroth exercises combined with sling training, Schroth exercises alone, conventional rehabilitation, and orthotic treatment ranked relatively high, whereas core stability training, digital therapeutics, and proprioceptive neuromuscular facilitation ranked relatively low. In direct comparisons, only Schroth exercises showed a statistically significant advantage over proprioceptive neuromuscular facilitation. This suggests that improvement in trunk rotational deformity may depend more on training methods specifically targeting the three-dimensional abnormalities of AIS (28).

Evaluation of AIS treatment efficacy should not only focus on imaging and body surface morphological changes, but also on patients' quality of life. Postural abnormalities caused by scoliosis, exercise limitations, and the long-term treatment burden may all affect patients' subjective health status (33). Therefore, this study included the SRS-22 score as an important outcome indicator. The SRS-22 comprehensively reflects the overall quality of life of AIS patients across multiple dimensions, including function, pain, self-image, mental health, and treatment satisfaction. The results of this study showed that, in terms of improvement in SRS-22 scores, Schroth exercises combined with sling training, Schroth exercises alone, and conventional rehabilitation ranked relatively high, whereas Schroth combined with postural re-education and pelvic rotation correction, proprioceptive neuromuscular facilitation, and core stability training ranked relatively low. Although the 95% confidence intervals of pairwise comparisons among all interventions crossed 0 and no clear statistical differences were identified, the probability ranking results suggest that some combined interventions centered on Schroth exercises still show certain potential for improving overall quality of life.

Previous evidence syntheses have generally supported the value of conservative exercise-based treatment for AIS. Ma et al. (10) found in a systematic review and meta-analysis that PSSE may improve Cobb angle and related clinical outcomes in children and adolescents with idiopathic scoliosis. Zhou et al. (11), in an overview of systematic reviews and meta-analyses, likewise concluded that exercise therapy is generally beneficial for AIS, although the included evidence showed substantial heterogeneity. These findings are broadly consistent with our results showing relatively favorable performance of scoliosis-specific exercise approaches. More specifically, Jiménez-Jiménez et al. (28) summarized that the Schroth method may improve Cobb angle, ATR, pulmonary function, and health-related quality of life. This is in line with our finding that Schroth-based interventions ranked relatively favorably across Cobb angle, ATR, and SRS-22. By contrast, Liu et al. (12) and Li et al. (16) reported beneficial effects of core stabilization or core-based exercise on scoliosis-related outcomes, whereas in our study these interventions did not consistently rank among the most favorable options. One possible explanation is that Schroth- or PSSE-based approaches more directly target the three-dimensional nature of AIS deformity, whereas general core training may have a stronger effect on trunk stability than on deformity-specific correction. In addition, the SOSORT guidelines proposed by Negrini et al. (7) emphasized that orthotic treatment and scoliosis-specific exercise are both key components of conservative management during growth. Our finding that brace-centered combined interventions showed relatively high ranking probabilities is broadly consistent with this recommendation. However, unlike previous reviews and guidelines, the present study simultaneously compared both single and combined conservative interventions within one network meta-analytic framework and across three clinically relevant dimensions, namely Cobb angle, ATR, and SRS-22. This broader comparison may provide additional evidence for individualized multimodal conservative treatment planning in AIS.

The strengths of this study lie in its relatively systematic inclusion of randomized controlled trials on both single and combined conservative interventions for AIS, and in its simultaneous evaluation of efficacy from three dimensions—Cobb angle, angle of trunk rotation, and SRS-22 score—making the findings more consistent with actual clinical needs. In addition, this study adopted a network meta-analysis approach, which can simultaneously integrate direct and indirect comparison evidence and rank the relative efficacy of different interventions, thereby providing a relatively comprehensive evidence-based basis for the development of individualized conservative treatment strategies in clinical practice.

This study has several limitations that should be considered when interpreting the findings. First, some intervention nodes, particularly Schroth combined with hippotherapy and certain brace-centered combined strategies, were informed by only a small number of studies with limited sample sizes. This may have reduced the stability of the SUCRA rankings and increased the possibility that the apparent advantages of these interventions were influenced by sparse data or individual small trials. Therefore, the favorable rankings of these interventions should not be interpreted as robust evidence of superiority. Second, substantial clinical heterogeneity existed across the included trials in terms of intervention duration, treatment frequency, follow-up time, and the content of routine care. Such heterogeneity may have weakened the comparability of studies and reduced the reliability of indirect comparisons within the network, which may partly explain why most pairwise comparisons did not reach statistical significance. Third, because blinding is difficult to implement in exercise- and brace-based trials, performance bias may have been unavoidable, particularly for subjective outcomes such as SRS-22, potentially leading to overestimation of patient-reported benefits. Fourth, the asymmetry observed in some funnel plots suggests possible small-study effects or publication bias, which may have led to an overrepresentation of positive findings and inflated the ranking probabilities of some interventions. Fifth, the present network meta-analysis should not be interpreted as a comprehensive comparison of all PSSE schools. Although PSSE refers to a broad category of scoliosis-specific exercise approaches, some approaches, such as BSPTS, DoboMed, FITS, and the Side-Shift Method, were absent or underrepresented in the eligible RCT network. Separating identifiable PSSE approaches, such as Schroth and SEAS, improves clinical specificity but may reduce precision due to smaller node sizes, whereas pooling all PSSE approaches could obscure clinically meaningful differences. Thus, the present node structure balances clinical homogeneity and statistical precision. Accordingly, rankings of sparsely represented nodes and their generalization to the broader PSSE field should be interpreted cautiously. Finally, no subgroup analyses were performed according to curve severity, curve pattern, or skeletal maturity. As a result, the present findings mainly reflect average comparative effects at the overall AIS population level and cannot be used to determine which intervention is optimal for specific clinical subgroups.

## 5 Conclusion

This study compared the relative efficacy of different single and combined conservative interventions for adolescent idiopathic scoliosis through a systematic review and network meta-analysis. The results showed that some combined conservative interventions ranked relatively high in terms of probability for outcomes such as Cobb angle, angle of trunk rotation, and SRS-22 scores. Schroth-based interventions ranked favorably for some outcomes, but these findings should be interpreted cautiously given the limited representation of some PSSE approaches in the eligible RCT network. Overall, combined interventions may be more consistent with the clinical characteristics of AIS, in which three-dimensional deformity coexists with functional impairment, but their relative advantages still require further confirmation through more large-sample, high-quality randomized controlled trials. In clinical practice, individualized conservative treatment strategies should be developed according to the severity of scoliosis, functional status, and patient compliance.

## Data Availability

The raw data supporting the conclusions of this article will be made available by the authors, without undue reservation.
