# Supplementary material for: Relative efficacy of different single and combined conservative interventions for adolescent idiopathic scoliosis: a systematic review and network meta-analysis
Source: Front Public Health. 2026 Oct 14;14:1860459. doi: 10.3389/fpubh.2026.1860459 (PMC13713608; doi:10.3389/fpubh.2026.1860459)
Supplement: Supplementary file 1 [file Table_1.docx]

**Appendix: Detailed Search Strategies for Each Database**

**Search period:** From database inception to December 31, 2025. No language restrictions were applied. The database-specific strategies below explicitly report the controlled-vocabulary terms (where applicable), exact free-text terms, Boolean operators, truncation/wildcard use, search fields, filters, date limits, and randomized controlled trial (RCT) search filters for each database, with platform-specific syntax provided to permit independent replication.

**1. PubMed**

• Search date limit: inception to 2025-12-31

• Search fields: MeSH Terms, Title/Abstract

• Controlled vocabulary: "Scoliosis"[MeSH], "Exercise Therapy"[MeSH], "Orthotic Devices"[MeSH], "Physical Therapy Modalities"[MeSH], and "Rehabilitation"[MeSH].

• Free-text terms: exactly as listed in search sets #2 and #5 below; no additional free-text terms were used.

• Boolean operators and truncation: OR was used within concept blocks, AND between population/intervention/study-design blocks, and NOT only for animal-only exclusion; * was used in "physical activit*" and "exercise therap*".

• Filters: animal-only records were excluded using #8–#9; no language filter was applied.

• RCT filter: search set #7 (randomized/controlled trial publication types plus random, randomly, trial, and RCT in Title/Abstract), combined with #9.

#1 "Scoliosis"[Mesh]
#2 "adolescent idiopathic scoliosis"[Title/Abstract] OR AIS[Title/Abstract] OR idiopathic scoliosis[Title/Abstract]
#3 #1 OR #2
#4 "Exercise Therapy"[Mesh] OR "Orthotic Devices"[Mesh] OR "Physical Therapy Modalities"[Mesh] OR "Rehabilitation"[Mesh]
#5 exercise[Title/Abstract] OR "physical activit*"[Title/Abstract] OR "physical exercise"[Title/Abstract] OR "exercise therap*"[Title/Abstract] OR orthotic[Title/Abstract] OR brace[Title/Abstract] OR braces[Title/Abstract] OR bracing[Title/Abstract] OR "orthotic treatment"[Title/Abstract] OR Schroth[Title/Abstract] OR SEAS[Title/Abstract] OR PSSE[Title/Abstract] OR "physiotherapeutic scoliosis-specific exercise"[Title/Abstract] OR Pilates[Title/Abstract] OR "core stabilization"[Title/Abstract] OR "core stability"[Title/Abstract] OR "home exercise"[Title/Abstract] OR hippotherapy[Title/Abstract] OR "balance training"[Title/Abstract] OR "suspension training"[Title/Abstract] OR "postural response correction"[Title/Abstract] OR "non-surgical treatment"[Title/Abstract] OR conservative[Title/Abstract]
#6 #4 OR #5
#7 randomized controlled trial[Publication Type] OR controlled clinical trial[Publication Type] OR random[Title/Abstract] OR randomly[Title/Abstract] OR trial[Title/Abstract] OR RCT[Title/Abstract]
#8 animals[MeSH] NOT humans[MeSH]
#9 #7 NOT #8
#10 #3 AND #6 AND #9

**2. Embase (via Elsevier)**

• Search date limit: inception to 2025-12-31

• Search fields: Emtree terms, title, abstract, keywords

• Controlled vocabulary (Emtree): 'idiopathic scoliosis'/exp, 'exercise therapy'/exp, 'physical activity'/exp, 'orthosis'/exp, 'brace'/exp, 'physical therapy'/exp, and 'rehabilitation'/exp.

• Free-text terms: exactly as listed in search sets #2 and #5 below in title, abstract, and keyword fields; no additional free-text terms were used.

• Boolean operators and truncation: OR was used within concept blocks and AND between concept blocks; * was used in 'physical activit*' and 'exercise therap*'.

• Filters: human studies were restricted using [humans]/lim in #9; no language filter was applied.

• RCT filter: search set #7 ('randomized controlled trial'/exp, 'randomization'/exp, random, randomly, trial, and rct), combined in #8.

#1 'idiopathic scoliosis'/exp
#2 'adolescent idiopathic scoliosis':ti,ab,kw OR AIS:ti,ab,kw OR 'idiopathic scoliosis':ti,ab,kw
#3 #1 OR #2
#4 'exercise therapy'/exp OR 'physical activity'/exp OR 'orthosis'/exp OR 'brace'/exp OR 'physical therapy'/exp OR 'rehabilitation'/exp
#5 exercise:ti,ab,kw OR 'physical activit*':ti,ab,kw OR 'physical exercise':ti,ab,kw OR 'exercise therap*':ti,ab,kw OR orthotic:ti,ab,kw OR brace:ti,ab,kw OR braces:ti,ab,kw OR bracing:ti,ab,kw OR 'orthotic treatment':ti,ab,kw OR schroth:ti,ab,kw OR seas:ti,ab,kw OR psse:ti,ab,kw OR 'physiotherapeutic scoliosis-specific exercise':ti,ab,kw OR pilates:ti,ab,kw OR 'core stabilization':ti,ab,kw OR 'core stability':ti,ab,kw OR 'home exercise':ti,ab,kw OR hippotherapy:ti,ab,kw OR 'balance training':ti,ab,kw OR 'suspension training':ti,ab,kw OR 'postural response correction':ti,ab,kw OR 'non-surgical treatment':ti,ab,kw OR conservative:ti,ab,kw
#6 #4 OR #5
#7 'randomized controlled trial'/exp OR 'randomization'/exp OR random:ti,ab,kw OR randomly:ti,ab,kw OR trial:ti,ab,kw OR rct:ti,ab,kw
#8 #3 AND #6 AND #7
#9 [humans]/lim AND #8

**3. Web of Science Core Collection**

• Search date limit: inception to 2025-12-31

• Search fields: Topic (TS = title, abstract, author keywords, Keywords Plus)

• Controlled vocabulary: not applicable; this Web of Science strategy used Topic free-text searching rather than a thesaurus/controlled-vocabulary field.

• Free-text terms: exactly as listed in the three TS= blocks below; no additional free-text terms were used.

• Boolean operators and truncation: OR was used within each concept block and AND between the population, intervention, and RCT blocks; * was used in "physical activit*" and "exercise therap*".

• Filters: no language or human/animal filter was applied.

• RCT filter: the third TS= block (random OR randomly OR trial OR "controlled trial" OR "randomized controlled trial" OR RCT).

TS=(("adolescent idiopathic scoliosis" OR AIS OR "idiopathic scoliosis"))
AND TS=((exercise OR "physical activit*" OR "physical exercise" OR "exercise therap*" OR orthotic OR brace OR braces OR bracing OR "orthotic treatment" OR Schroth OR SEAS OR PSSE OR "physiotherapeutic scoliosis-specific exercise" OR Pilates OR "core stabilization" OR "core stability" OR "home exercise" OR hippotherapy OR "balance training" OR "suspension training" OR "postural response correction" OR "non-surgical treatment" OR conservative))
AND TS=((random OR randomly OR trial OR "controlled trial" OR "randomized controlled trial" OR RCT))

**4. Scopus**

• Search date limit: inception to 2025-12-31

• Search fields: TITLE-ABS-KEY

• Controlled vocabulary: not applicable; this Scopus strategy used TITLE-ABS-KEY free-text searching rather than a thesaurus/controlled-vocabulary field.

• Free-text terms: exactly as listed in the three TITLE-ABS-KEY blocks below; no additional free-text terms were used.

• Boolean operators and truncation: OR was used within each concept block and AND between the population, intervention, and RCT blocks; * was used in "physical activit*" and "exercise therap*".

• Filters: no language or human/animal filter was applied.

• RCT filter: the third TITLE-ABS-KEY block (random OR randomly OR trial OR "controlled trial" OR "randomized controlled trial" OR RCT).

TITLE-ABS-KEY(("adolescent idiopathic scoliosis" OR AIS OR "idiopathic scoliosis"))
AND TITLE-ABS-KEY((exercise OR "physical activit*" OR "physical exercise" OR "exercise therap*" OR orthotic OR brace OR braces OR bracing OR "orthotic treatment" OR Schroth OR SEAS OR PSSE OR "physiotherapeutic scoliosis-specific exercise" OR Pilates OR "core stabilization" OR "core stability" OR "home exercise" OR hippotherapy OR "balance training" OR "suspension training" OR "postural response correction" OR "non-surgical treatment" OR conservative))
AND TITLE-ABS-KEY((random OR randomly OR trial OR "controlled trial" OR "randomized controlled trial" OR RCT))

**5. Cochrane Library (CENTRAL)**

• Search date limit: inception to 2025-12-31

• Search fields: MeSH descriptor, title, abstract, keywords

• Controlled vocabulary (MeSH): [Scoliosis], [Exercise Therapy], [Orthotic Devices], [Physical Therapy Modalities], and [Rehabilitation], each exploded as shown below.

• Free-text terms: exactly as listed in search sets #2 and #8 below in title, abstract, and keyword fields; no additional free-text terms were used.

• Boolean operators and truncation: OR was used within concept blocks and AND between the population, intervention, and RCT blocks; * was used in "physical activit*" and "exercise therap*".

• Filters: no language or additional human/animal filter was applied.

• RCT filter: search set #10 (random OR randomly OR trial OR "controlled trial" OR "randomized controlled trial" OR RCT), combined in #11.

#1 MeSH descriptor: [Scoliosis] explode all trees
#2 ("adolescent idiopathic scoliosis" OR AIS OR "idiopathic scoliosis"):ti,ab,kw
#3 #1 OR #2
#4 MeSH descriptor: [Exercise Therapy] explode all trees
#5 MeSH descriptor: [Orthotic Devices] explode all trees
#6 MeSH descriptor: [Physical Therapy Modalities] explode all trees
#7 MeSH descriptor: [Rehabilitation] explode all trees
#8 (exercise OR "physical activit*" OR "physical exercise" OR "exercise therap*" OR orthotic OR brace OR braces OR bracing OR "orthotic treatment" OR Schroth OR SEAS OR PSSE OR "physiotherapeutic scoliosis-specific exercise" OR Pilates OR "core stabilization" OR "core stability" OR "home exercise" OR hippotherapy OR "balance training" OR "suspension training" OR "postural response correction" OR "non-surgical treatment" OR conservative):ti,ab,kw
#9 #4 OR #5 OR #6 OR #7 OR #8
#10 (random OR randomly OR trial OR "controlled trial" OR "randomized controlled trial" OR RCT):ti,ab,kw
#11 #3 AND #9 AND #10
